# Intimate partner violence against ever-partnered women in Europe: Prevalence and associated factors—Results from the violence against women EU-wide survey

**DOI:** 10.3389/fpubh.2022.1033465

**Published:** 2022-12-02

**Authors:** Alice Barbier, Patrick Chariot, Thomas Lefèvre

**Affiliations:** Institut de recherche interdisciplinaire sur les enjeux sociaux, UMR8156 CNRS – U997 Inserm – EHESS – USPN, Aubervilliers, France

**Keywords:** associated factors, Europe, ever-partnered women, intimate partner violence, partner, prevalence

## Abstract

**Study questions:**

To describe the prevalence of physical, sexual, and psychological intimate partner violence (IPV) against women in the European Union (EU) and to search for their determinants among demographic, socioeconomic, health-related factors, and partner characteristics.

**Methods:**

Observational study. Data from the violence against women survey, the first study conducted in the EU, which simultaneously measured all dimensions of IPV and many characteristics. The EU Agency for Fundamental Rights randomly conducted face-to-face interviews among the 28 countries with 42,002 women aged 18–74 who resided in the survey country and spoke the language. IPV is defined by a positive answer to at least one question about physical, sexual, or psychological violence perpetrated by a current or ex-partner.

**Findings:**

Among the 40,357 women having already been in a relationship, 51.7% (51.2–52.2) reported having been victims of violence in their lifetime. The prevalence of physical, sexual, and psychological IPV was, respectively, 20.0% (19.6–20.4), 8.4% (8.2–8.7), and 48.5% (48.1–49.0). Women, who were younger, employed, had highly qualified work, had at least one immigrant parent, lived in an urban setting, were unmarried, separated, divorced, widowed, childless, cohabited with a partner, and others over the age of 18, had worse self-perceived health, or a history of violence before the age of 15 were more likely to report IPV. It was the same when their partners had a lower level of education, no work, were home staying, earned less than they did, were involved in 10 years of relationship, were frequently drunk, or were violent otherwise.

**Major implication:**

The lifetime prevalence of reported IPV among women in Europe is high and likely underestimated. The results emphasize the importance of a comprehensive definition of IPV and partners' characteristics. They highlight socioeconomic differences and poorer health status for victims of IPV.

## Introduction

Not only does intimate partner violence (IPV) against women constitute a major violation of women's rights, but it also is a public health problem. IPV refers to behavior by an intimate partner or ex-partner that causes physical, sexual, or psychological harm, including physical aggression, sexual coercion, psychological abuse, and controlling behaviors (1). More than a third of women's homicides would be caused by IPV (2). IPV causes physical injuries, which at times may require surgical management (3). From a psychological standpoint, IPV is associated with incident depressive symptoms and suicide attempts (4), post-traumatic stress disorder (5), and an increase in drug use (6). IPV is also significantly associated with HIV (7). Women who have experienced violence would also be more exposed to cardiovascular diseases (8) and have molecular alteration causing, in general, greater long-term morbidity (9). Furthermore, IPV exposition is gradually recognized as a form of child abuse, which harms the health of children who witness violence (10). IPV against women is a European issue (11), although obviously present internationally (12). Recently, the issue of IPV has had all the media coverage during the COVID-19 pandemic, particularly with the lockdown measures (13). In 2013, the World Health Organization (WHO) compiled evidence on the lifetime prevalence of physical or sexual IPV, which has been estimated at 25.4% among European women (14). Risk factors seemed to be identified, such as young age, low education level, unemployment, low income, acculturation, the experience of child abuse, divorced status, and cohabitation (15). Available European data about IPV are derived from several, usually asynchronous and heterogeneous studies (Appendix A). The definition of IPV varies across studies, and its measure is often partial, that is, considering only one or two dimensions. Sexual and psychological IPV are often excluded from analyses. Although one study explored up to 18 potential determinants, most studies consider a much more limited number of determinants at the same time. To date, no large-scale study has been conducted in the European Union (EU) that consistently and simultaneously measured all physical, sexual, and psychological dimensions of IPV and a broad range of demographic, socioeconomic, and health-related characteristics. The present study aimed to describe the prevalence of physical, sexual, psychological, and overall IPV against exposed women in the EU general population and searched for their determinants among demographic, socioeconomic, health-related factors, and partner characteristics.

## Materials and methods

### Study design and participants

Data came from the violence against women EU-wide survey (VAWS), a multi-country, population-based, and cross-sectional study conducted by the EU Agency for Fundamental Rights (FRA) (16). A questionnaire was developed over 2 years by the FRA survey team composed of established academic experts and practitioners in the field of violence against women. To develop the questionnaire, FRA referred to the International Violence Against Women Survey and the WHO multi-country study on women's health and domestic violence. Details of the methods and response calculations have been described elsewhere (17). A random sample was compiled using addresses of households across the 28 member states of the EU using a two-stage clustered stratified randomization design. Primary sampling units (PSUs) were selected with probability proportional to size: wherever possible, local electoral territorial units were used as PSUs. Census enumeration districts or local authorities were used as PSUs where local electoral territorial units were available. Samples were stratified by geographical region and by urban or rural character. Then a geographical cluster sampling occurred. Addresses were randomly selected in each cluster. The survey was introduced at the door about women's wellbeing and safety. All women aged 18–74 years living under the same roof in the household who could speak one of the official languages of the country were eligible. Only women away from the household for 3 months or longer were excluded. A contact sheet was developed to document the respondent selection process and ensure that households and individuals were randomly selected.

### Patient and public involvement

Agency for Fundamental Rights carried out a pilot study in six countries in 2011 (17). This one was designed to test the draft survey through the use of face-to-face interviews and focus group discussions. Interviewers were trained to carry out interviews with randomly selected women (15 respondents in each country) and with women who were identified by women's shelters and other victim support organizations as having been victims of violence (10 respondents in each country). Attention was also given to having women from different age groups participate. The interviews were based on a questionnaire that the interviewer and the interviewee completed together, followed by a cognitive interview. The cognitive interviews explored different women's understanding of key concepts to be examined. They related, in particular, to experiences of physical, sexual, and psychological violence in the intimate sphere as well as new settings, such as internet-based social networks. The interviews with women identified by women's shelters and other support organizations were recorded. Researchers listening to the recorded interviews used and agreed the criteria to code instances where the interviewee had problems answering a particular item or asked for more information. Following the results of the pretest, FRA revised the draft survey questionnaire before it was used. The results highlighted the role of the associations as a resource for interviewees who would like to continue talking about their experiences with someone after the survey.

### Procedures

Physical, sexual, and psychological IPV were defined by a positive answer to at least one question about, respectively, physical, sexual, or psychological violence perpetrated by a current or previous partner (Appendix B). IPV was defined as the manifestation of at least one type of physical, sexual, or psychological IPV. To identify items corresponding to IPV and choose which category each item belonged to, that is, physical, sexual, or psychological IPV, we used the WHO definition (18). This definition only gives four examples of physical violence, two of sexual violence, and six of psychological violence whereas we had 52 items to classify (Appendix B). When it was not sufficiently precise, we used the Center for Disease Control and Prevention definition, which gives a more exhaustive definition of IPV, six pages long (19). Other variables were relative to women (eight variables to demographic and socioeconomic status, six to marital status and household composition, and five to health) and their partners when they had one at the time of the study (eight variables). Violence before the age of 15 was defined by a positive answer to at least one question about violence before the age of 15 (Appendix C). It was considered domestic if the father, mother, brother, or sister perpetrated it. Trained female interviewers did the measurement of all the variables. In-person and face-to-face interviews in interviewees' homes were conducted using the standard questionnaire translated into the main languages used in the EU member states. The interviewers had to have a minimum of 3 months of experience in random probability survey work and the ability to follow precise instructions. They could decline to work on the project if they were not comfortable with the topic. Training for interviewers focused on discussion of the random methodology and how to deal with distressed respondents. Households had to be located by the interviewer with women after a minimum of three visits or calls and when information about the household was not refused by the first contact.

### Statistical methods

Numeric variables were categorized. The R® 3.6.0 software was used. We used the frequency function for proportions and the Wald method for their 95% confidence interval (95% CI). Associations were analyzed by bivariable logistic regressions. Three models of multivariable logistic regressions were run to control for confounding: demographic and socioeconomic model (Model 1); marital status, couple, and home-related model (Model 2); and health-related model (Model 3). Factors related to women were explored only among women in couples or having a previous couple relationship and referred to lifetime IPV. A multivariable logistic regression model was run, including only variables related to current partners, only among women in couples, and referred to current IPV. All multivariable models were adjusted on age. The odds ratio (OR) and their 95% CI were obtained with a tidy function. Each result was weighted by svydesign and svrepdesign functions. All factors and models were analyzed in each group of victims of overall physical, sexual, or psychological violence. Missing data (MD) were counted as a category.

## Results

### Participants

Agency for Fundamental Rights collected data between March and September 2012. Eligible women were 77,109. The questionnaire response rate was 42.1% (17). Less than 1% of people contacted were unable to take part because they did not speak one of the official languages. The statements of 42,023 women were collected, and 21 of them were removed from the dataset at the data cleaning stage. Among 42,002 statements, 31,222 women (74%) reported being in a relationship, and 26,765 (64%) reported having been at least once in a relationship.

### Descriptive data

#### Demographic data

Women were 30% to be over the age of 60, and 70% lived in an urban area (Table 1). At least one of their parents was an immigrant for 12% of them. The majority of them were married or in a civil partnership (60%) and had children (75%).

**Table 1:** General characteristics of ever-partnered whole population and victims and non-victims of intimate partner violence.

	**All women**		**Non-IPV**		**IPV**		**OR**	**(95% CI)**
	***N*** **= 40,357**	**(%)**	***N*** **= 19,485**	**(%)**	***N*** **= 20,872**	**(%)**		
**Age**								
**MD (*****N*** **=** **65)**								
18–29	7,528	(18.7)	3,130	(16.1)	4,398	(21.1)	Ref	
30–39	7,992	(19.8)	3,484	(17.9)	4,508	(21.6)	0.92	(0.82–1.03)
40–49	8,368	(20.7)	4,075	(20.9)	4,293	(20.6)	0.75	(0.66–0.85)
50–59	7,574	(18.8)	3,850	(19.8)	3,724	(17.8)	0.69	(0.60–0.78)
≥60	8,829	(21.9)	4,918	(25.2)	3,912	(18.7)	0.57	(0.50–0.64)
**Highest level of education**								
**MD (*****N*** **=** **148)**								
Primary	14,791	(36.7)	7,663	(39.3)	7,128	(34.2)	Ref	
Secondary	17,335	(43.0)	8,090	(41.5)	9,245	(44.3)	1.23	(1.14–1.33)
Tertiary	8,083	(20.0)	3,665	(18.8)	4,418	(21.2)	1.30	(1.18–1.42)
**Employment status**								
**MD (*****N*** **=** **131)**								
Paid work	21,071	(52.2)	9,575	(49.1)	11,496	(55.1)	Ref	
Retired	6,825	(16.9)	3,773	(19.4)	3,053	(14.6)	0.67	(0.61–0.74)
Homemaker	5,326	(13.2)	2,968	(15.2)	2,359	(11.3)	0.66	(0.59–0.74)
Student, in training	2,439	(6.0)	1,097	(5.6)	1,342	(6.4)	1.02	(0.83–1.26)
Unemployed	3,472	(8.6)	1,566	(8.0)	1,907	(9.1)	1.01	(0.88–1.17)
Other	1,093	(2.7)	418	(2.1)	674	(3.2)	1.34	(1.08–1.67)
Employment during past 12 months	14,702	(36.4)	1,778	(9.1)	2,452	(11.7)	1.62	(1.41–1.86)
**MD (*****N*** **=** **21,425)**								
**Occupation type**								
**MD (*****N*** **=** **258)**								
Employed	16,708	(41.4)	7,901	(40.5)	8,807	(42.2)	Ref	
Farmer or fisherwomen	816	(2.0)	446	(2.3)	370	(1.8)	0.74	(0.55–1.00)
Professional	1,462	(3.6)	615	(3.2)	847	(4.1)	1.24	(1.02–1.51)
Owner	2,265	(5.6)	1,073	(5.5)	1,192	(5.7)	1.00	(0.84–1.18)
Management	4,874	(12.1)	2,267	(11.6)	2,608	(12.5)	1.03	(0.92–1.16)
Supervisor	266	(0.7)	101	(0.5)	165	(0.8)	1.47	(0.96–2.26)
Manual worker/servant	10,162	(25.2)	4,807	(24.7)	5,355	(25.7)	1.00	(0.91–1.10)
Never done paid work	3,546	(8.8)	2,126	(10.9)	1,420	(6.8)	0.60	(0.52–0.68)
Citizen	39,006	(96.7)	18,949	(97.2)	20,057	(96.1)	0.69	(0.56–0.87)
**MD (*****N*** **=** **46)**								
At least one migrated parent	4,844	(12.0)	2,026	(10.4)	2,818	(13.5)	1.48	(1.14–1.93)
**MD (*****N*** **=** **442)**								
**Type of locality**								
**MD (*****N*** **=** **226)**								
Urban	28,082	(69.6)	12,997	(66.7)	15,085	(72.3)	Ref	
Rural	12,049	(29.9)	6,354	(32.6)	5,696	(27.3)	0.62	(0.55–0.69)
Married or in a civil partnership	24,754	(61.3)	13,420	(68.9)	11,333	(54.3)	0.54	(0.49–0.58)
**MD (*****N*** **=** **114)**								
Cohabitation with a partner	26,286	(65.1)	13,352	(68.5)	12,993	(62.0)	0.75	(0.68–0.82)
**MD (*****N*** **=** **487)**								
**Children**								
MD (*N* = 47)	30,234	(74.9)	16,246	(83.4)	13,988	(67.0)	0.40	(0.37–0.44)
**Number of people living in the household**								
**Under the age of 18**								
**MD (*****N*** **=** **872)**								
0	21,982	(54.5)	10,333	(53.0)	11,650	(55.8)	Ref	
1	8,075	(20.0)	3,969	(20.4)	4,106	(19.7)	0.92	(0.84–1.00)
2 or more	9,429	(23.4)	4,810	(24.7)	4,619	(22.1)	0.85	(0.78–0.93)
**Aged 18 and over**								
**MD (*****N*** **=** **336)**								
1	6,507	(16.1)	2,592	(13.3)	3,915	(18.8)	Ref	
2 or more	33,514	(83.0)	16,745	(85.9)	16,769	(80.3)	0.66	(0.60–0.73)
**Last relationship**								
**MD (*****N*** **=** **3,114)**								
No	13,402	(33.2)	9,363	(47.0)	4,239	(20.3)	Ref	
Separation	16,697	(41.4)	6,008	(30.8)	10,688	(51.2)	3.85	(3.55–4.17)
Divorce	4,291	(10.6)	964	(4.9)	3,327	(15.9)	7.46	(6.50–8.57)
Widowhood	2,854	(7.1)	1,604	(8.2)	1,250	(6.0)	1.68	(1.48–1.92)
**Perceived healthiness**								
**MD (*****N*** **=** **26)**								
Good	29,095	(72.1)	14,595	(74.9)	14,500	(69.5)	Ref	
Fair	8,466	(21.0)	3,778	(19.4)	4,688	(22.5)	1.25	(1.14–1.37)
Bad	2,770	(6.9)	1,101	(5.7)	1,669	(8.0)	1.53	(1.31–1.78)
Limitation	5,583	(13.8)	2,186	(11.2)	3,397	(16.3)	1.54	(1.40–1.70)
**MD (*****N*** **=** **70)**								
Perceived disability	1,823	(4.5)	678	(3.5)	1,145	(5.5)	1.14	(0.92–1.42)
**MD (*****N*** **=** **34,863)**								
Violence before the age of 15	14,343	(35.5)	4,858	(24.9)	9,465	(45.4)	2.53	(2.33–2.75)
**MD (*****N*** **=** **37)**								
Domestic violence before the age of 15	11,442	(28.4)	3,718	(19.1)	7,724	(37.0)	2.52	(2.31–2.74)
**MD (*****N*** **=** **37)**								

Source: FRA Violence Against Women Survey dataset, 2012.

IPV, being a victim of intimate partner violence; OR, odds ratio; CI, confidence interval; MD, missing data.

IPV population is compared to the non-IPV population for each variable (e.g., age), and results are given by OR.

For each variable, the reference category is indicated as “Ref” (e.g., for age, the 18–29 age class is the category of reference for OR).

When not specified, “no” is used as a reference.

OR computed with logistic regression models.

MD was considered as a category.

#### Socioeconomic data

Most women reported a secondary level of education (43%). The majority had paid work (52%, mainly with an employed status), and 36% were unemployed during the past year.

#### Health-related data

A majority of women (69%) considered their health to be good, and only 5% perceived a disability. They reported having experienced child abuse in 36% of cases.

#### Partners-related data

When women had current partners, those were 23% to be over the age of 60 (Table 2). A majority of women reported that their partner had a secondary level of education (69%) and paid work (69%), mainly with an employed status. A majority of women reported that their partner earned more than them (61%). Most reported that their partner was not drunk more than once a month (93%). Only 7% of them declared that their partner was violent outside the family.

**Table 2:** General characteristics of current partners of the whole population and victims and non-victims of intimate partner violence.

	**All current partners**		**Non-IPV**		**IPV**		**OR**	**(95% CI)**
	***N*** **= 31,222**	**(%)**	***N*** **= 20,199**	**(%)**	***N*** **= 11,023**	**(%)**		
**Age**								
**MD (*****N*** **=** **990)**								
18–29	3,963	(12.7)	2,166	(10.7)	1,797	(16.3)	Ref	
30–39	6,056	(19.4)	3,640	(18.0)	2,417	(21.9)	0.80	(0.69–0.93)
40–49	6,800	(21.8)	4,607	(22.8)	2,193	(19.9)	0.57	(0.48–0.68)
50–59	6,208	(19.9)	4,202	(20.8)	2,007	(18.2)	0.58	(0.49–0.67)
≥60	7,205	(23.1)	4,735	(23.4)	2,470	(22.4)	0.63	(0.53–0.74)
**Highest level of education**								
**MD (*****N*** **=** **1,251)**								
Primary	2,352	(7.5)	1,438	(7.1)	914	(8.3)	Ref	
Secondary	21,664	(69.4)	13,905	(68.8)	7,759	(70.4)	0.88	(0.76–1.01)
Tertiary	5,956	(19.1)	3,841	(19.0)	3,115	(19.3)	0.87	(0.73–1.03)
**Employment status**								
**MD (*****N*** **=** **1,041)**								
Paid work	21,465	(68.7)	13,798	(68.3)	7,666	(69.5)	Ref	
Retired	5,639	(18.1)	3,656	(18.1)	1,983	(18.0)	0.98	(0.88–10.8)
Homemaker	149	(0.5)	69	(0.3)	80	(0.7)	2.08	(1.20–3.63)
Student, in training	843	(2.7)	520	(2.6)	323	(2.9)	1.12	(0.81–1.55)
Unemployed	1,416	(4.5)	843	(4.2)	573	(5.2)	1.22	(1.00–1.50)
Other	669	(2.1)	417	(2.1)	251	(2.3)	1.08	(0.84–1.39)
**Occupation type**								
**MD (*****N*** **=** **1,188)**								
Employed	7,081	(22.7)	4,566	(22.6)	2,515	(22.8)	Ref	
Farmer or fisherwomen	900	(2.9)	568	(2.8)	332	(3.0)	1.06	(0.81–1.39)
Professional	1,365	(4.4)	846	(4.2)	518	(4.7)	1.11	(0.93–1.32)
Owner	3,149	(10.1)	2,020	(10.0)	1,130	(10.2)	1.02	(0.88–1.17)
Management	5,044	(16.2)	3,243	(16.1)	1,801	(16.3)	1.01	(0.88–1.15)
Supervisor	656	(2.1)	448	(2.2)	209	(1.9)	0.85	(0.61–1.17)
Manual worker/servant	11,184	(35.8)	7,103	(35.2)	4,081	(37.0)	1.04	(0.94–1.16)
Never done paid work	655	(2.1)	428	(2.1)	227	(2.1)	0.96	(0.70–1.34)
**Drunkenness**								
**MD (*****N*** **=** **1,364)**								
Less than a couple of times a month	28,955	(92.7)	18,713	(92.6)	10,242	(92.9)	Ref	
One or two times a week	674	(2.2)	304	(1.5)	371	(3.4)	2.23	(1.71–2.90)
Every day	229	(0.7)	66	(0.3)	163	(1.5)	4.53	(3.01–6.80)
Violence outside the family	2,205	(7.1)	1,012	(5.0)	1,213	(11.0)	2.32	(1.94–2.77)
**MD (*****N*** **=** **2,024)**								
**Earning**								
**MD (*****N*** **=** **2,719)**								
Roughly the same than woman	5,864	(18.8)	3,770	(18.7)	2,093	(19.0)	Ref	
More than woman	19,179	(61.4)	12,416	(61.5)	6,763	(61.4)	0.98	(0.88–1.09)
Less than woman	3,461	(11.1)	2,001	(9.9)	1,460	(13.2)	1.31	(1.13–1.53)
**Length of relation with woman**								
**MD (*****N*** **=** **1,518)**								
< 1 year	1,074	(3.4)	743	(3.7)	331	(3.0)	Ref	
1–10 years	9,504	(30.4)	5,085	(25.2)	4,419	(40.1)	1.95	(1.42–2.69)
11–20 years	6,112	(19.6)	4,138	(20.5)	1,974	(17.9)	1.07	(0.78–1.48)
20–30 years	5,260	(16.8)	3,655	(18.1)	1,605	(14.6)	0.99	(0.71–1.37)
>30 years	7,755	(24.8)	5,419	(26.8)	2,336	(21.2)	0.97	(0.70–1.34)

Source: FRA Violence Against Women Survey dataset, 2012.

IPV, being the current partner of a victim of intimate partner violence; OR, odds ratio; CI, confidence interval; MD, missing data.

IPV current partner population is compared to the non-IPV current partner population for each variable (e.g., age), and results are given by OR.

For each variable, the reference category is indicated as “Ref” (e.g., for age, the 18–29 age class is the category of reference for OR).

When not specified, “no” is used as a reference.

OR computed with logistic regression models.

MD was considered as a category.

### Outcome data

#### Lifetime prevalence of IPV

The lifetime prevalence of overall reported IPV was 51.7% (95% CI 51.2–52.2) (Table 3). The prevalence of physical, sexual, and psychological IPV was, respectively, 20.0% (19.6–20.4), 8.4% (8.2–8.7), and 48.5% (48.1–49.0). Women could have experienced only one or two of the three IPV dimensions during their life or all three. Women were 6.3% to report having experienced all three (Table 4). The most prevalent form of isolated IPV was psychological IPV (29.6%), and isolated physical or sexual IPV was reported in < 1% of cases. Cumulated physical and psychological IPV was the most two-dimensional IPV reported (12.8%). Psychological IPV was the most prevalent isolated reported type of IPV (29.6%). Physical, respectively sexual IPV was about 3-fold, respectively about 4-fold less reported when the declared perpetrator was the current partner (respectively 7.0% for the current partner vs. 24.1% for the previous partner and 2.3 vs. 10.7%).

**Table 3:** Prevalence of lifetime physical, sexual, psychological, and overall intimate partner violence perpetrated by a previous or current partner.

	**Current partner**			**Previous partner**			**Current or previous partner**		
	***N*** **= 31,222**	**(%)**	**(95% CI)**	***N*** **= 26,765**	**(%)**	**(95% CI)**	***N*** **= 40,357**	**(%)**	**(95% CI)**
Physical IPV	2,178	(7.0)	(6.7–7.2)	6,439	(24.1)	(23.5–24.6)	8,075	(20.0)	(19.6–20.4)
	MD (*N* = 941)			MD (*N* = 732)			MD (*N* = 20,262)		
Sexual IPV	717	(2.3)	(2.1–2.5)	2,866	(10.7)	(10.3–11.1)	3,404	(8.4)	(8.2–8.7)
	MD (*N* = 1,806)			MD (*N* = 1,461)			MD (*N* = 22,693)		
Psychological IPV	10,720	(34.3)	(33.8–34.9)	13,469	(50.3)	(49.7–50.9)	20,429	(48.5)	(48.1–49.0)
	MD (*N* = 5,992)			MD (*N* = 8,884)			MD (*N* = 17,558)		
Physical, sexual, or psychological IPV	11,023	(35.3)	(34.8–35.8)	13,803	(51.6)	(51.0–52.2)	20,872	(51.7)	(51.2–52.2)
	MD (*N* = 5,926)			MD (*N* = 8,673)			MD (*N* = 17,185)		

Source: FRA Violence Against Women Survey dataset, 2012.

IPV, intimate partner violence; OR, odds ratio; CI, confidence interval; MD, missing data.

**Table 4:** Lifetime prevalence of all combinations of reported types of intimate partner violence (psychological, physical, and sexual) in ever-partnered women (*n* = 40,357).

	**Physical violence**		**Sexual violence**		**Psychological violence**	
	***N*** **= 40,357**	**(%)**	***N*** **= 40,357**	**(%)**	***N*** **= 40,357**	**(%)**
Physical violence	358	(0.9)	10	(0.0)	5,158	(12.8)
Sexual violence			75	(0.2)	769	(1.9)
Psychological violence					11,952	(29.6)
	***N*** **=** **40,357**	**(%)**				
Physical, sexual, and psychological violence	2,549	(6.3)				

*Source*. FRA Violence Against Women Survey dataset, 2012.

The table reads as a matric, for example, 1.9% of ever-partnered women reported having experienced both IPV sexual and psychological violence in their lifetime.

The last line indicates that 6.3% of ever-partnered women experienced sexual, psychological, and physical IPV in their lifetime.

#### Bivariable associations between IPV and characteristics of women and partners

##### Demographic data

Women who reported lifetime IPV had more likely at least one immigrant parent and were more likely separated or divorced (Table 1). Conversely, women who reported no lifetime IPV were more than the others over 40 years old, more often a citizen of the country where they reside, living in rural areas, married or civil partnered, having children, and cohabiting with their partner, several children, or others over the age of 18.

##### Socioeconomic data

Women who reported lifetime IPV had more likely a higher level of education and an intellectual profession and were more likely employed during the past 12 months (Table 1). Conversely, women who reported no lifetime IPV were more than the others who retired or were homemakers and had less often been in paid employment.

##### Health-related data

Women who reported lifetime IPV were more likely to perceive their health as worse, to declare more often a disability, and to have been victims of violence before the age of 15 (Table 1).

##### Partner-related data

When they reported IPV perpetrated by their current partner, this partner was more often than the others < 40 years old, a homemaker, drunk more than once a week or every day, violent otherwise, having a lower earning, and engaged in a relationship of 1–10 years (Table 2). When they declared less current IPV, their partners were more than the others, over 40 years old.

##### Direction and magnitude of effects

Associations between IPV and women's age, current marital status, having children, and composition of the household not reduced to the couple were not consistent according to which IPV dimension was considered and could be of opposite effects. The magnitudes of associations (OR, 95% CI) ranged from 1.24 (1.14–1.33, secondary level of education) to 7.46 (6.50–8.57, divorce as a mode of ending the last relationship) for women's characteristics and from 1.31 (1.13–1.53, partner earning less that woman) to 4.53 (3.01–6.80, partner everyday drunkenness) for partner characteristics. Results of bivariable models for physical, sexual, and psychological IPV are reported in Appendix D–I.

#### Multivariable associations between IPV and characteristics of women and partners

##### Demographic data

Overall reported IPV was associated with the following women characteristics: being younger, having at least one immigrant parent, living in an urban setting, being unmarried, cohabiting with a partner, or others over the age of 18, being childless, and having been separated, divorced, or widowed (Table 5).

**Table 5:** Associations between overall, physical, sexual, and psychological intimate partner violence (IPV) and ever-partnered women characteristics.

	**Overall IPV**		**Physical IPV**		**Sexual IPV**		**Psychological IPV**	
	**OR**	**(95% CI)**	**OR**	**(95% CI)**	**OR**	**(95% CI)**	**OR**	**(95% CI)**
**Model 1: Demographic and socioeconomic status**								
**Age**								
18–29	Ref		Ref		Ref		Ref	
30–39	0.84	(0.73–0.97)	1.16	(0.93–1.44)	1.33	(0.98–1.80)	0.85	(0.73–0.98)
40–49	0.69	(0.60–0.79)	1.26	(1.03–1.54)	1.53	(1.14–2.07)	0.68	(0.59–0.79)
50–59	0.64	(0.56–0.73)	1.04	(0.86–1.28)	1.21	(0.89–1.64)	0.62	(0.54–0.72)
≥ 60	0.58	(0.49–0.68)	0.96	(0.75–1.22)	1.22	(0.82–1.81)	0.58	(0.49–0.68)
**Highest level of education**								
Primary	Ref		Ref		Ref		Ref	
Secondary	1.04	(0.96–1.12)	0.83	(0.74–0.94)	0.82	(0.70–0.96)	1.06	(0.97–1.15)
Tertiary	0.98	(0.87–1.10)	0.69	(0.59–0.80)	0.73	(0.58–0.91)	0.98	(0.86–1.12)
**Employment status**								
Paid work	Ref		Ref		Ref		Ref	
Retired	0.77	(0.51–1.18)	0.65	(0.35–1.21)	0.61	(0.25–1.46)	0.81	(0.50–1.30)
Homemaker	0.68	(0.45–1.02)	0.71	(0.41–1.23)	0.73	(0.33–1.62)	0.70	(0.44–1.11)
Student, in training	0.77	(0.49–1.21)	0.71	(0.39–1.28)	0.69	(0.29–1.64)	0.83	(0.52–1.33)
Unemployed	0.84	(0.55–1.28)	0.89	(0.51–1.55)	0.78	(0.36–1.69)	0.88	(0.57–1.35)
Other	1.38	(0.90–2.11)	1.28	(0.75–2.18)	1.75	(0.83–3.70)	1.42	(0.93–2.17)
Employment during past 12 months	1.23	(1.06–1.42)	1.19	(1.02–1.40)	1.20	(0.93–1.56)	1.22	(1.06–1.41)
**Occupation type**								
Employed	Ref		Ref		Ref		Ref	
Farmer or fisherwomen	1.00	(0.80–1.25)	1.57	(1.12–2.21)	2.38	(1.32–4.28)	0.99	(0.77–1.27)
Professional	1.25	(1.02–1.52)	1.08	(0.86–1.36)	1.16	(0.79–1.69)	1.28	(1.04–1.57)
Owner	0.05	(0.86–1.29)	1.09	(0.88–1.35)	1.09	(0.82–1.43)	1.07	(0.91–1.26)
Management	1.09	(0.97–1.23)	1.08	(0.90–1.28)	1.21	(0.95–1.54)	1.09	(0.96–1.23)
Supervisor	1.53	(1.06–2.20)	1.56	(0.94–2.60)	1.93	(1.00–3.69)	1.60	(1.07–2.38)
Manual worker/servant	1.04	(0.94–1.14)	1.14	(1.02–1.27)	1.21	(1.01–1.44)	1.05	(0.96–1.14)
Never done paid work	0.64	(0.53–0.77)	0.61	(0.48–0.77)	0.62	(0.44–0.88)	0.64	(0.54–0.76)
Citizen	0.92	(0.69–1.23)	0.91	(0.68–1.22)	1.01	(0.65–1.57)	0.88	(0.69–1.13)
At least one migrated parent	1.23	(1.07–1.41)	1.26	(1.10–1.45)	1.30	(1.05–1.62)	1.22	(1.07–1.40)
**Type of locality**								
Urban	Ref		Ref		Ref		Ref	
Rural	0.81	(0.75–0.86)	0.70	(0.37–1.42)	0.76	(0.66–0.87)	0.80	(0.74–0.87)
**Model 2: Marital status and household**								
**Age**								
18–29	Ref		Ref		Ref		Ref	
30–39	1.19	(1.01–1.40)	0.98	(0.79–1.22)	1.20	(0.90–1.59)	1.19	(1.03–1.37)
40–49	1.05	(0.90–1.23)	1.01	(0.83–1.24)	1.34	(0.99–1.80)	1.04	(0.90–1.20)
50–59	1.10	(0.94–1.29)	0.98	(0.79–1.21)	1.24	(0.89–1.71)	1.06	(0.91–1.24)
≥60	1.01	(0.84–1.22)	0.92	(0.74–1.14)	1.22	(0.81–1.84)	1.00	(0.86–1.16)
Married or in a civil partnership	0.55	(0.47–0.64)	0.73	(0.63–0.84)	0.64	(0.49–0.83)	0.54	(0.47–0.63)
Cohabitation with a partner	3.08	(2.55–3.71)	1.18	(0.98–1.41)	1.15	(0.87–1.52)	3.06	(2.55–3.68)
Children	0.37	(0.33–0.43)	1.50	(1.24–1.81)	1.49	(1.18–1.89)	0.37	(0.32–0.43)
**Number of people living in the household**								
**Under the age of 18**								
0	Ref		Ref		Ref		Ref	
1	1.01	(0.89–1.14)	1.15	(0.98–1.34)	0.98	(0.78–1.23)	1.01	(0.89–1.15)
2 or more	0.97	(0.86–1.09)	1.17	(1.02–1.34)	1.25	(0.96–1.63)	0.96	(0.85–1.08)
**Aged 18 and over**								
1	Ref		Ref		Ref		Ref	
2 or more	0.71	(0.61–0.84)	0.76	(0.65–0.87)	0.87	(0.68–1.10)	0.72	(0.61–0.86)
**Last relationship**								
No	Ref				Ref		Ref	
Separation	3.53	(3.19–3.89)	4.72	(4.05–5.50)	5.37	(4.09–7.07)	3.05	(3.17–3.89)
Divorce	9.24	(7.92–10.78)	10.07	(8.41–12.07)	9.53	(7.04–12.91)	9.30	(8.04–10.76)
Widowhood	2.25	(1.90–2.66)	2.55	(1.94–3.37)	3.19	(2.18–4.67)	2.25	(1.92–2.64)
**Model 3: Health**								
**Age**								
18–29	Ref		Ref		Ref		Ref	
30–39	0.83	(0.73–0.95)	1.08	(0.89–1.32)	1.26	(0.95–1.65)	0.83	(0.72–0.95)
40–49	0.62	(0.55–0.70)	1.05	(0.88–1.24)	1.28	(0.99–1.67)	0.61	(0.53–0.70)
50–59	0.53	(0.46–0.60)	0.80	(0.68–0.95)	0.96	(0.73–1.25)	0.51	(0.44–0.58)
≥60	0.40	(0.35–0.45)	0.60	(0.51–0.71)	0.77	(0.57–1.04)	0.39	(0.34–0.45)
**Perceived healthiness**								
Good	Ref		Ref		Ref		Ref	
Fair	1.39	(1.26–1.54)	1.66	(1.46–1.88)	1.67	(1.40–2.00)	1.39	(1.26–1.53)
Bad	1.59	(1.32–1.91)	1.99	(1.64–2.42)	1.77	(1.28–2.46)	1.57	(1.34–1.85)
Limitation	1.91	(0.90–4.04)	2.63	(1.15–6.05)	4.19	(1.51–11.67)	1.88	(0.94–3.77)
Perceived disability	1.04	(0.86–1.27)	0.96	(0.77–1.20)	0.95	(0.67–1.35)	1.02	(0.87–1.21)
Violence before the age of 15	2.04	(1.77–2.36)	2.17	(1.85–2.56)	2.36	(1.86–3.00)	1.99	(1.74–2.29)
Domestic violence before the age of 15	1.35	(1.16–1.57)	1.35	(1.14–1.60)	1.36	(1.05–1.75)	1.37	(1.18–1.60)

Source: FRA Violence Against Women Survey dataset, 2012.

OR, odds ratio; CI, confidence interval; MD, missing data.

When not specified, “no” is used as a reference.

OR computed with multinomial logistic regression models.

Three models were built as follows: Model 1 based on sociodemographic data; Model 2 based on marital status and constitution of the household; and Model 3 based on self-perceived health and history of violence.

All models were adjusted on age.

Victims of each type of IPV (e.g., overall IPV, sexual IPV) are compared to non-victims considered the same type of IPV for each variable (e.g., age), and results are given by OR.

For each variable, the reference category is indicated as “Ref”.

MD was considered as a category.

##### Socioeconomic data

Overall reported IPV was associated with being employed and having highly qualified work (Table 5).

##### Health-related data

Overall reported IPV was associated with having worse self-perceived health and reporting a history of violence before the age of 15 (Table 5).

##### Partner-related data

Overall, IPV was also associated with several partner characteristics: lower level of education, not working or home staying, earning less than the woman, engagement in a relationship of 1–10 years, frequency of drunkenness, and being violent otherwise (Table 6).

**Table 6:** Associations between overall, physical, sexual, and psychological intimate partner violence (IPV) and characteristics of current partners of IPV victims.

	**Overall IPV**		**Physical IPV**		**Sexual IPV**		**Psychological IPV**	
	**OR**	**(95% CI)**	**OR**	**(95% CI)**	**OR**	**(95% CI)**	**OR**	**(95% CI)**
**Age**								
18–29	Ref		Ref		Ref		Ref	
30–39	0.83	(0.69–1.00)	1.30	(0.90–1.86)	4.50	(0.09–218.85)	0.81	(0.67–0.98)
40–49	0.70	(0.58–0.85)	1.37	(0.91–2.06)	5.74	(0.07–435.56)	0.68	(0.56–0.84)
50–59	0.86	(0.70–1.05)	1.41	(0.91–2.17)	7.99	(0.10–637.01)	0.84	(0.68–1.04)
≥60	0.92	(0.72–1.18)	1.57	(0.87–2.84)	14.58	(0.24–900.54)	0.90	(0.70–1.17)
**Highest level of education**								
Primary	Ref		Ref		Ref		Ref	
Secondary	0.82	(0.69–0.98)	0.56	(0.42–0.74)	0.44	(0.28–69.26)	0.84	(0.70–1.00)
Tertiary	0.78	(0.63–0.97)	0.43	(0.31–0.61)	0.23	(0.16–3.31)	0.81	(0.64–1.01)
**Employment status**								
Paid work	Ref		Ref		Ref		Ref	
Retired	1.31	(1.13–1.52)	1.02	(0.70–1.48)	0.63	(0.24–1.65)	1.32	(1.14–1.53)
Homemaker	1.91	(1.03–3.55)	3.03	(1.35–6.79)	2.66	(0.15–45.79)	1.76	(0.98–3.17)
Student, in training	0.89	(0.53–1.48)	1.28	(0.49–3.35)	0.54	-	0.87	(0.52–1.45)
Unemployed	1.09	(0.88–1.35)	1.47	(1.10–1.97)	1.07	(0.55–2.08)	1.10	(0.88–1.37)
Other	1.15	(0.86–1.54)	1.32	(0.83–2.10)	1.49	(0.66–3.38)	1.15	(0.86–1.55)
**Occupation type**								
Employed	Ref		Ref		Ref		Ref	
Farmer or fisherwomen	1.13	(0.89–1.44)	1.89	(1.16–3.10)	3.99	(0.57–28.07)	1.13	(0.89–1.43)
Professional	1.20	(0.94–1.52)	1.19	(0.77–1.86)	2.97	(0.47–18.96)	1.22	(0.96–1.55)
Owner	1.13	(0.97–1.32)	1.17	(0.86–1.61)	2.33	(0.52–10.44)	1.12	(0.95–1.31)
Management	1.17	(1.01–1.35)	1.11	(0.85–1.46)	1.44	(0.19–11.12)	1.15	(0.99–1.33)
Supervisor	0.83	(0.58–1.18)	0.47	(0.22–1.01)	2.09	(0.42–10.32)	0.86	(0.61–1.23)
Manual worker/servant	1.02	(0.90–1.16)	0.95	(0.77–1.17)	1.28	(0.36–4.56)	1.04	(0.92–1.18)
Never done paid work	0.85	(0.52–1.41)	0.66	(0.22–1.97)	1.36	(0.01–2.66)	0.87	(0.53–1.44)
**Earning**								
Roughly the same than woman	Ref		Ref		Ref		Ref	
More than woman	0.99	(0.88–1.12)	1.14	(0.93–1.40)	0.98	(0.62–1.57)	0.99	(0.87–1.12)
Less than woman	1.28	(1.09–1.50)	1.24	(0.85–1.79)	1.33	(0.66–2.68)	1.25	(1.06–1.47)
**Length of the relation with woman**								
< 1 year	Ref		Ref		Ref		Ref	
1–10 years	1.95	(1.44–2.64)	0.88	(0.42–1.85)	0.52	(0.12–2.26)	1.95	(1.44–2.65)
11–20 years	1.09	(0.80–1.49)	1.07	(0.49–2.33)	0.36	(0.09–1.45)	1.08	(0.78–1.48)
21–30 years	0.99	(0.71–1.40)	1.14	(0.50–2.59)	0.21	(0.04–1.03)	0.96	(0.68–1.36)
>30 years	0.75	(0.54–1.04)	0.89	(0.40–2.00)	0.20	(0.03–98.94)	0.72	(0.51–1.00)
**Drunkenness**								
Less than a couple of times a month	Ref		Ref		Ref		Ref	
One or two times a week	1.65	(1.49–2.55)	3.13	(2.25–4.34)	2.62	(1.25–5.49)	1.97	(1.51–2.57)
Every days	4.32	(2.76–6.76)	8.70	(5.73–13.20)	7.58	(2.10–27.34)	4.34	(2.80–6.74)
Violence outside the family	2.22	(1.90–2.59)	4.62	(3.73–5.72)	5.95	(0.95–37.11)	2.19	(1.88–2.54)

Source: FRA Violence Against Women Survey dataset, 2012.

OR, odds ratio; CI, confidence interval; MD, missing data.

When not specified, “no” is used as the reference.

OR computed with multinomial logistic regression models.

Current partners of victims of each type of IPV (e.g., overall IPV, sexual IPV) are compared to current partners of non-victims considered the same type of IPV for each variable (e.g., age), and results are given by OR.

For each variable, the reference category is indicated as “Ref” (e.g., for age, the 18–29 age class is the category of reference for OR).

MD were considered as a category.

##### Associations by type of violence

Never having done paid work and being married or civil partnered were significantly associated with less lifetime reported IPV, regardless of the type of IPV (Table 5). Having at least one immigrant parent, being separated, divorced, or widowed, a worse perceived health, and reporting a history of violence before the age of 15 was significantly associated with more lifetime reported IPV but also the frequency of partner drunkenness, regardless of the type of IPV (Table 6). Women aged 40–49, as well as those who had children, declared significantly more physical or sexual lifetime IPV and conversely less psychological lifetime IPV (Table 5).

#### Links between health and IPV

Regarding health, there was a positive and significant association between IPV and women's poor perceived health (OR close to 1.50 with regard to all dimensions of IPV), as well as a history of violence before the age of 15 (domestic or otherwise), regardless of the type of IPV (Table 5). Physical and sexual IPV were also associated with more women's limitations. There was a positive association between each type of IPV and partner drunkenness and between overall physical and psychological IPV and partner violence otherwise (Table 6).

## Discussion

### Key results

European women reported a high lifetime prevalence of IPV: 51.7% for overall IPV and 29.6% for isolated psychological IPV. Psychological IPV aside, most cases were two or three-dimensional IPV that combined physical and psychological (12.8%) or physical, psychological, and sexual dimensions (6.3%). The major component of the 20.0% prevalence of physical IPV was associated with at least another type of IPV when isolated physical IPV represented only 0.9%. Prevalence varied systematically according to whether the reported assailant was the current or a former partner: reported prevalence was lower when the perpetrator was the current partner, especially for physical (7.0 vs. 24.1%) and sexual (2.3 vs. 10.7%) IPV. All 27 screened characteristics of women or current partners were associated with at least one IPV type or overall IPV. Among the 19 women's characteristics, 13 remained after adjustments, and nine were consistently associated with all IPV types and overall IPV. Bad self-perceived health and functional limitation were associated with reported IPV.

### Limitations

The roles of age, marital status, and cohabitation with the current partner are unclear. While being older was associated with more reported physical IPV, being younger was associated with more psychological, sexual, and overall IPV. We could not state whether age exposure to IPV is associated with being more aware and prone to disclose IPV. Being married or in a civil partnership was consistently associated with less reported IPV across all types of IPV: whether marriage is protective against IPV while being partially a condition of exposure or a condition that prevents women from reporting IPV could not be determined here (20). Cohabitation with a current partner could be associated with less or more IPV, depending on whether analyses were adjusted or not. Telephone recruitment of randomly selected women was used in countries with long distances between addresses and low population density (Denmark, Finland, and Sweden). It can have lowered the overall response rate. The cultural settings, acceptability of unsolicited approaches, and saturation with other surveys could also have an effect on the resulting response rate. The subject of violence against women, because of the general level of interest in the topic, perceived relevance of the survey, and experience of women could have affected the response rate. The latter was close to that observed in the European Working Conditions Survey (48%) (21). Standardization of the questionnaire and interviewers' training prevented observer bias during data collection. The memorization effort and subjectivity of some women's responses could have led to a recall bias. A misestimation of IPV prevalence is possible, notably sexual or psychological IPV, for which some items are less objective and may be influenced by cultural settings. However, two recent studies supported the comparability of data between countries (22). The interview setting in the women's home with the possible violent partner's presence could have decreased violence statements. The high difference between the prevalence of violence perpetrated by the ex-partner and by the current partner could be explained by a relative over-declaration of the ex-partner perpetrated IPV and a relative under-reporting of violence perpetrated by the current partner. There is no easy explanation for the current partner to be less violent than the previous partner. The existence of several ex-partners compared to a single current one may downplay the differences, but this does not seem to be the main source of the differences. On the other hand, women can talk less about it. This could mean that statements about the current partner are of limited value due to unspeakable violence. Despite a large number of variables for adjustment, we cannot rule out the possibility of some residual confounding. We lack information about the individual level (psychiatric history as antisocial behavior, traditional genre role attitudes, substance use as marijuana or tobacco), relational level (conjugal conflicts, pregnancy period, women's family structure as single parent, women's family education level and socioeconomic status, partner experience of parental monitoring), and community level (laws, cultural acceptance of IPV, media IPV normalization, social support, and neighborhood disadvantage). We have highlighted the current characteristics of women who have experienced IPV in their lifetime, that is, cumulative IPV. However, we do not know how they differ from those who were victims at the time of the investigation, which limits our understanding of their link to violence. Furthermore, we have studied physical, sexual, and psychological violence separately without exploring the possibility of another typology of violence explored in the study of Podaná (23). The need to differentiate between different types of VC was emphasized by Nevala, particularly in terms of coercion (24).

### Important findings

#### Prevalence of IPV among European women

To the best of our knowledge, VAWS is the largest population-based survey on IPV among European women, which provides an acceptable statistical power. The findings are strengthened by the exhaustive, standardized, and concurrent measure of items. Our results are consistent with compiled results of the WHO report on European global and regional estimates of violence against women, which is 25.4% of the lifetime prevalence of physical or sexual IPV among ever-partnered women (14). The COVID-19 pandemic, associated in particular with successive lockdown periods, had a probable impact on the IPV. However, we do not know if the prevalence would have increased if the study had been conducted during or after this period (25).

#### Links between socioeconomic position and IPV

Our results support the view that IPV would be related to socioeconomic position, which was previously pointed out by Reichel (26). As was found by Costa et al. for physical IPV in Europe, manual work was associated with all types of IPV victimization (27). Similarly, the partner's unemployment was associated with more physical IPV perpetration. Our findings that age and being married are associated with less IPV are similar to results reported previously in a meta-analysis of prospective-longitudinal studies by Yakubovich et al. (28). Older women may report less IPV. However, the link between young age and IPV seems relevant as it exists even though it is a cumulative lifetime IPV. Our results, therefore, highlight the relevance of implementing preventive measures for young women and men that help reduce IPV. In addition, one study found that sexual harassment disproportionately affected younger women (29). Furthermore, this study showed, like our study concerning IPV, that women in higher occupational groups were more affected by sexual harassment. This suggests that younger age and higher occupations would put women at greater risk of violence, in general. Women not cohabiting with a partner and engaged in a recent couple relationship also appear to be more specifically exposed to IPV. Our findings that education level is a significant protective factor against IPV were not found for women in the meta-analyze of Yakubovich et al. (28). Analysis of VAWS data study among young women does not show any association between educational level and IPV (30). Because of the transversal design of the VAWS, we cannot establish temporality between education level and IPV. The COVID-19 pandemic-related lockdown has likely had unprecedented negative economic impacts and exacerbated some of the risk factors for IPV, including job loss and reduced income (31).

#### Links between a history of violence, health, and IPV

In the same way, causality cannot be demonstrated between women's perceived health, the partner's regular drunkenness, and the perpetration of violence outside the family. Further explorations might be necessary that follow women and partners throughout their lifetime and analyze changes in life factors and IPV in a repeated-measures design. As modifiable factors, their improvement could help decrease IPV. These characteristics could inversely help to identify IPV risk groups. Till-Tentschert suggested that women who have experienced violence before the age of 15 were at greater risk of experiencing physical and sexual abuse in later life (32). The strength of association estimated in our study and respect for temporality despite transversal design strengthens this idea. However, child abuse was not found to be a significant risk factor for IPV in the meta-analysis by Yakubovich et al., where retrospective histories of child abuse were excluded (28), and the association in our study was significant for physical and sexual IPV but not significant for overall and psychological IPV when child abuse was domestic. Further studies about revictimization are needed to explain the involvement of child abuse in the different types of IPV.

#### Specificity of psychological violence

As was the case of domestic child abuse, limitation, or presence of several majors at household, physical and sexual IPV had the most common factors. It does suggest common mechanisms, distinct from psychological IPV. Furthermore, psychological IPV seems to be mainly single perpetrated, whereas physical and sexual IPV seems to be associated with psychological IPV in most cases. Given the less important number of studies about psychological IPV than physical IPV in literature, despite the high prevalence of psychological IPV, further studies about psychological IPV may be required for understanding IPV.

### Generalizability

The characteristics of the sample are consistent with European data for age in 2019 in particular (10.8% age group 15–24 and 14.3% age group 65–79) (33). However, some women, such as homeless persons and those living in institutions such as nursing homes, prisons, army barracks, or student hostels, were not included. Non-inclusion of students living in hostels may explain the width of the confidence interval concerning students. An underestimation of OR in this population is possible because of the protective character of age and marriage for overall IPV and the presence of several adults in the household for physical and sexual IPV. Further investigation is needed to characterize the risk in the student population. This is especially so because the student status of the partner is significantly associated with overall and psychological IPV in this study.

## Conclusion

While our findings in terms of IPV prevalence and associated factors are mainly confirmatory of what can be found in the literature (34–36), our study presents at least four main interests that make it important. First, this survey is the first and only survey that has been conducted in Europe at such a scale, simultaneously across 28 countries with the same unique method. Second, it brings robust evidence that IPV is mainly a combination of at least two types of violence and is seldomly the experience of a single type of violence. Third, we show that reported prevalences dramatically vary according to whether the perpetrator is a current or an ex-partner, inviting researchers to find ways to control for divulgations biases in further studies that would be related to this characteristic. Fourth, the partner data reported here make this paper unique, especially since the information comes from a large sample (be it the women reporting and not the men). The results apply only to women and do not fully describe IPV as a whole, which may concern men. Physical IPV declared by men was estimated at 3.8% as victims and 10.0% as perpetrators and victims at once in Europe by Costa et al. (27). This study should not be used to suggest that studies and interventions need to be only targeted at women.

## Data availability statement

Publicly available datasets were analyzed in this study. This data can be found here: https://ukdataservice.ac.uk/.

## Author contributions

PC and TL were responsible for the conceptualization of the study and obtained access to data. TL designed the study and was responsible for the statistical design and oversight. AB and TL verified the underlying data and did the literature search. AB analyzed data. AB, TL, and PC wrote the manuscript. All authors had access to all the data and had responsibility for data interpretation, drafting, revision, and final approval of the manuscript's submission for publication.

## Conflict of interest

The authors declare that the research was conducted in the absence of any commercial or financial relationships that could be construed as a potential conflict of interest.

## Publisher's note

All claims expressed in this article are solely those of the authors and do not necessarily represent those of their affiliated organizations, or those of the publisher, the editors and the reviewers. Any product that may be evaluated in this article, or claim that may be made by its manufacturer, is not guaranteed or endorsed by the publisher.

## Abbreviations

CI: Confidence interval
EU: European Union
FRA: Agency for Fundamental Rights
IPV: Intimate partner violence
MD: Missing data
OR: Odds ratio
PSUs: Primary sampling units
WHO: World Health Organization.

## References

[1] World Health Organisation. Violence Against Women. (2021). Available online at: https://www.who.int/news-room/fact-sheets/detail/violence-against-women (accessed November 23, 2022).

[2] Stöckl H Devries K Rotstein A Abrahams N Campbell J Watts C. The global prevalence of intimate partner homicide: a systematic review. Lancet. (2013) 382:859–65. 10.1016/S0140-6736(13)61030-2 23791474

[3] Sprague S Madden K Dosanjh S Godin K Goslings JC Schemitsch EH. Intimate partner violence and musculoskeletal injury: bridging the knowledge gap in orthopaedic fracture clinics. BMC Musculoskelet Disord. (2013) 14:23. 10.1186/1471-2474-14-23 23316813 PMC3585708

[4] Devries KM Mak JY Bacchus LJ Child JC Falder G Petzold M. Intimate partner violence and incident depressive symptoms and suicide attempts: a systematic review of longitudinal studies. PLoS Med. (2013) 10:1001439. 10.1371/journal.pmed.1001439 23671407 PMC3646718

[5] Machisa MT Christofides N Jewkes R. Mental ill health in structural pathways to women's experiences of intimate partner violence. PLoS ONE. (2017) 12:0175240. 10.1371/journal.pone.0175240 28384241 PMC5383260

[6] Peters EN Khondkaryan E Sullivan TP. Associations between expectancies of alcohol and drug use, severity of partner violence, and posttraumatic stress among women. J Interpers Violence. (2012) 27:2108–27. 10.1177/0886260511432151 22258078 PMC3646585

[7] Li Y Marshall CM Rees HC Nunez A Ezeanolue EE Ehiri JE. Intimate partner violence and HIV infection among women: a systematic review and meta-analysis. J Int AIDS Soc. (2014) 17:18845. 10.7448/IAS.17.1.18845 24560342 PMC3925800

[8] Halpern LR Shealer ML Cho R McMichael EB Rogers J Ferguson-Young D. Influence of intimate partner violence (IPV) exposure on cardiovascular and salivary biosensors: is there a relationship? J Natl Med Assoc. (2017) 109:252–61. 10.1016/j.jnma.2017.08.001 29173932 PMC7388656

[9] Humphreys J Epel ES Cooper BA Lin J Blackburn EH Lee KA. Telomere shortening in formerly abused and never abused women. Biol Res Nurs. (2012) 14:115–23. 10.1177/1099800411398479 21385798 PMC3207021

[10] McTavish JR MacGregor JCD Wathen CN MacMillan HL. Children's exposure to intimate partner violence: an overview. Int Rev Psychiatry. (2016) 28:504–18. 10.1080/09540261.2016.1205001 27414209

[11] Gracia E. Intimate partner violence against women and victim-blaming attitudes among Europeans. Bull World Health Organ. (2014) 92:380–1. 10.2471/BLT.13.131391 24839328 PMC4007130

[12] García-Moreno C Zimmerman C Morris-Gehring A Heise L Amin A Abrahams N. Addressing violence against women: a call to action. Lancet. (2015) 385:1685–95. 10.1016/S0140-6736(14)61830-4 25467579

[13] World Health Organization. COVID-19 and Violence Against Women: What the Health Sector/System Can Do. (2020). Available online at: https://apps.who.int/iris/handle/10665/331699 (accessed November 23, 2022).

[14] World Health Organization. Global and Regional Estimates of Violence Against Women. (2013). Available online at: http://www.who.int/reproductivehealth/publications/violence/9789241564625/en/ (accessed November 23, 2022).

[15] Pisinger C Døssing M. A systematic review of health effects of electronic cigarettes. Prev Med. (2014) 69:248–60. 10.1016/j.ypmed.2014.10.009 25456810

[16] European Union Agency for Fundamental Rights. Violence Against Women: An EU-Wide Survey. Main Results Report. (2014). Available online at: http://fra.europa.eu/en/publication/2014/violence-against-women-eu-wide-survey-main-results-report (accessed November 23, 2022).

[17] European Union Agency for Fundamental Rights. Violence Against Women: An EU-Wide Survey - Survey Methodology, Sample Fieldwork. Technical report (2014). Available online at: http://fra.europa.eu/en/publication/2014/violence-against-women-eu-wide-survey-survey-methodology-sample-and-fieldwork (accessed November 23, 2022).

[18] World Health Organization. Understanding and Addressing Violence Against Women. (2012). Available online at: https://apps.who.int/iris/bitstream/handle/10665/77432/WHO_RHR_12.36_eng.pdf (accessed November 23, 2022).

[19] *Centers for Disease Control and Prevention*. Intimate Partner Violence Surveillance : Uniform Definitions and Recommended Data Elements. Version 2.0 (2015). Available online at: https://stacks.cdc.gov/view/cdc/31292 (accessed November 23, 2022).

[20] Abbas J Aqeel M Ling J Ziapour A Raza MA Rehna T. Exploring the relationship between intimate partner abuses, resilience, psychological, and physical health problems in Pakistani married couples: a perspective from the collectivistic culture. Sex Relat Ther. (2020) 1–30. 10.1080/14681994.2020.1851673

[21] Eurofound. Fieldwork: Response Rate. (2011). Available online at: https://www.eurofound.europa.eu/surveys/ewcs/2005/responserate (accessed November 23, 2022).

[22] Gracia E Martín-Fernández M Lila M Merlo J Ivert AK. Prevalence of intimate partner violence against women in Sweden and Spain: a psychometric study of the ≪ Nordic paradox ≫. PLoS ONE. (2019) 14:0217015. 10.1371/journal.pone.0217015 31095614 PMC6522122

[23] Podaná Z. Patterns of intimate partner violence against women in Europe: prevalence and associated risk factors. J Epidemiol Community Health. (2021) 75:772–8. 10.1136/jech-2020-214987 33431512

[24] Nevala S. Coercive control and its impact on intimate partner violence through the lens of an EU-wide survey on violence against women. J Interpers Violence. (2017) 32:1792–820. 10.1177/0886260517698950 30156990

[25] Iverson KM Dardis CM Cowlishaw S Webermann AR Shayani DR Dichter ME. Effects of intimate partner violence during COVID-19 and pandemic-related stress on the mental and physical health of women veterans. J Gen Intern Med. (2022) 37:724–33. 10.1007/s11606-022-07589-z 36042090 PMC9427167

[26] Reichel D. Determinants of intimate partner violence in Europe: the role of socioeconomic status, inequality, and partner behavior. J Interpers Violence. (2017) 32:1853–73. 10.1177/0886260517698951 30156988

[27] Costa D Hatzidimitriadou E Ioannidi-Kapolou E Lindert J Soares J Sundin Ö. Intimate partner violence and health-related quality of life in European men and women: findings from the DOVE study. Qual Life Res. (2015) 24:463–71. 10.1007/s11136-014-0766-9 25063083

[28] Yakubovich AR Stöckl H Murray J Melendez-Torres GJ Steinert JI Glavin CEY. Risk and protective factors for intimate partner violence against women: systematic review and meta-analyses of prospective-longitudinal studies. Am J Public Health. (2018) 108:1–11. 10.2105/AJPH.2018.304428 29771615 PMC5993370

[29] Latcheva R. Sexual harassment in the european union: a pervasive but still hidden form of gender-based violence. J Interpers Violence. (2017) 32:1821–52. 10.1177/0886260517698948 30156991

[30] Sanz-Barbero B López Pereira P Barrio G Vives-Cases C. Intimate partner violence against young women: prevalence and associated factors in Europe. J Epidemiol Community Health. (2018) 72:611–6. 10.1136/jech-2017-209701 29519883

[31] Mahlangu P Gibbs A Shai N Machisa M Nunze N Sikweyiya Y. Impact of COVID-19 lockdown and link to women and children's experiences of violence in the home in South Africa. BMC Public Health. (2022) 22:1029. 10.1186/s12889-022-13422-3 35597933 PMC9123923

[32] Till-Tentschert U. The relation between violence experienced in childhood and women's exposure to violence in later life: evidence from Europe. J Interpers Violence. (2017) 32:1874–94. 10.1177/0886260517698952 30156989

[33] Eurostat. Statistics. (2022). Available online at: https://ec.europa.eu/eurostat/databrowser/view/tps00010/default/table?lang=en (accessed November 23, 2022).

[34] Garcia-Moreno C Jansen HAFM Ellsberg M Heise L Watts CH. WHO Multi-country Study on Women's Health and Domestic Violence against Women Study Team. Prevalence of intimate partner violence: findings from the WHO multi-country study on women's health and domestic violence. Lancet. (2006) 368:1260–9. 10.1016/S0140-6736(06)69523-8 17027732

[35] Devries KM Mak JYT García-Moreno C Petzold M Child JC Falder G. Global health. The global prevalence of intimate partner violence against women. Science. (2013) 340:1527–8. 10.1126/science.1240937 23788730

[36] Ismayilova L El-Bassel N. Prevalence and correlates of intimate partner violence by type and severity: population-based studies in Azerbaijan, Moldova, and Ukraine. J Interpers Violence. (2013) 28:2521–56. 10.1177/0886260513479026 23508086

