# Supplementary material for: Intimate partner violence against ever-partnered women in Europe: Prevalence and associated factors—Results from the violence against women EU-wide survey
Source: Front Public Health. 2022 Dec 2;10:1033465. doi: 10.3389/fpubh.2022.1033465 (PMC9755339; doi:10.3389/fpubh.2022.1033465)
Supplement: Supplementary file 1 [file Data_Sheet_1.docx]

Appendix A. Evidence before the study linked to Pubmed

| Fanslow J, Hashemi L, Malihi Z, Gulliver P, McIntosh T. Change in prevalence rates of physical and sexual intimate partner violence against women: data from two cross-sectional studies in New Zealand, 2003 and 2019. *BMJ Open,* 2021; 11:e044907. |
| --- |
| Kiwuwa-Muyingo S, Kadengye DT. Prevalence and risk factors for women's reports of past-year intimate partner violence: a comparative analysis of six east african national surveys. *J Interpers Violence* Published Online First: 1 November 2020. https://doi:10.1177/0886260520969374. |
| Ellsberg M, Ugarte W, Ovince J, Blackwell A, Quintanilla M. Long-term change in the prevalence of intimate partner violence: a 20-year follow-up study in León, Nicaragua, 1995-2016. *BMJ Glob Health,* 2020; 5:e002339. |
| Gibbs A, Dunkle K, Jewkes R. The prevalence, patterning and associations with depressive symptoms and self-rated health of emotional and economic intimate partner violence: a three-country population based study. *J Glob Health,* 2020; 10:010415. |
| Kwaramba T, Ye JJ, Elahi C, Lunyera J, Chotte Oliveira A, Sanches Calvo PR, et al. Lifetime prevalence of intimate partner violence against women in an urban Brazilian city: A cross-sectional survey. *PLoS One,* 2019; 14:e0224204. |
| Elghossain T, Bott T, Akik T, Obermeyer CM. Prevalence of intimate partner violence against women in the Arab world: a systematic review. *BMC Int Health Hum Rights,* 2019; 19:29. |
| Sanz-Barbero B, Barón N, Vives-Cases C. Prevalence, associated factors and health impact of intimate partner violence against women in different life stages. *PLoS One,* 2019; 14:e0221049. |
| An JH, Moon CS, Kim DE, Lee-Tauler SY, Jeon HJ, Cho SJ, et al. Prevalence of intimate partner violence victimization and its association with mental disorders in the Korean general population. *Arch Womens Ment Health,* 2019; 22:751-758. |
| Gracia E, Martín-Fernández M, Lila M, Merlo J, Ivert AK. Prevalence of intimate partner violence against women in Sweden and Spain: A psychometric study of the 'Nordic paradox'. *PLoS One,* 2019; 14:e0217015. |
| Bott S, Guedes A, Ruiz-Celis AP, Adams Mendoza J. Intimate partner violence in the Americas: a systematic review and reanalysis of national prevalence estimates. *Rev Panam Salud Publica,* 2019; 43:e26. |
| Memiah P, Ah Mu T, Prevot K, Cook CK, Mwangi MM, Mwangi EW, et al. The prevalence of intimate partner violence, associated risk factors, and other moderating effects: findings from the Kenya national health demographic survey. *J Interpers Violence* Published Online First: 12 October 2018. https://doi:10.1177/0886260518804177. |
| Hellmann DF, Kinninger MW, Kliem S. Sexual violence against women in Germany: prevalence and risk markers. *Int J Environ Res* *Public Health,* 2018; 15:1613. |
| Alangea DO, Addo-Lartey AA, Sikweyiya Y, Chirwa ED, Coker-Appiah D, Jewkes R, et al. Prevalence and risk factors of intimate partner violence among women in four districts of the central region of Ghana: Baseline findings from a cluster randomised controlled trial. *PLoS One,* 2018; 13:e0200874. |
| LaBore K, Ahmed T, Rizwan-Ur-Rashid, Ahmed R. Prevalence and Predictors of Violence Against Women in Pakistan. *J Interpers Violence* Published online first: 23 January 2019. https://doi.org/10.1177/0886260518824652. |
| Memiah P, Ah Mu T, Prevot K, Cook CK, Mwangi MM, Mwangi EW, et al. The Prevalence of Intimate Partner Violence, Associated Risk Factors, and Other Moderating Effects: Findings From the Kenya National Health Demographic Survey. *J Interpers Violence* Published online first: 12 October 2018. https://doi.org/10.1177/0886260518804177. |
| Ogum Alangea D, Addo-Lartey AA, Sikweyiya Y, Chirwa ED, Coker-Appiah D, Jewkes R, et al. Prevalence and risk factors of intimate partner violence among women in four districts of the central region of Ghana: Baseline findings from a cluster randomised controlled trial. *PLoS One,* 2018; 13:e0200874. |
| [Navarro-Mantas L](https://www.ncbi.nlm.nih.gov/pubmed/?term=Navarro-Mantas%20L%5BAuthor%5D&cauthor=true&cauthor_uid=29897003), [Velásquez MJ](https://www.ncbi.nlm.nih.gov/pubmed/?term=Vel%C3%A1squez%20MJ%5BAuthor%5D&cauthor=true&cauthor_uid=29897003), [Lemus S](https://www.ncbi.nlm.nih.gov/pubmed/?term=Lemus%20S%5BAuthor%5D&cauthor=true&cauthor_uid=29897003), [Megías JL](https://www.ncbi.nlm.nih.gov/pubmed/?term=Meg%C3%ADas%20JL%5BAuthor%5D&cauthor=true&cauthor_uid=29897003). Prevalence and Sociodemographic Predictors of Intimate Partner Violence Against Women in El Salvador. [*J Interpers Violence*](https://www.ncbi.nlm.nih.gov/pubmed/29897003) 2018; 36:NP3547-73. |
| Kassa GM, Abajobir AA. Prevalence of Violence Against Women in Ethiopia: A Meta-Analysis. *Trauma Violence Abuse,* 2020; 21:624-37. |
| [Chuemchit M](https://www.ncbi.nlm.nih.gov/pubmed/?term=Chuemchit%20M%5BAuthor%5D&cauthor=true&cauthor_uid=29904232), [Chernkwanma S](https://www.ncbi.nlm.nih.gov/pubmed/?term=Chernkwanma%20S%5BAuthor%5D&cauthor=true&cauthor_uid=29904232), [Rugkua R](https://www.ncbi.nlm.nih.gov/pubmed/?term=Rugkua%20R%5BAuthor%5D&cauthor=true&cauthor_uid=29904232), [Daengthern L](https://www.ncbi.nlm.nih.gov/pubmed/?term=Daengthern%20L%5BAuthor%5D&cauthor=true&cauthor_uid=29904232), [Abdullakasim P](https://www.ncbi.nlm.nih.gov/pubmed/?term=Abdullakasim%20P%5BAuthor%5D&cauthor=true&cauthor_uid=29904232), [Wieringa SE](https://www.ncbi.nlm.nih.gov/pubmed/?term=Wieringa%20SE%5BAuthor%5D&cauthor=true&cauthor_uid=29904232). Prevalence of Intimate Partner Violence in Thailand. [*J Fam* *Violence*](https://www.ncbi.nlm.nih.gov/pubmed/29904232), 2018; 33:315–323. |
| [Jewkes R](https://www.ncbi.nlm.nih.gov/pubmed/?term=Jewkes%20R%5BAuthor%5D&cauthor=true&cauthor_uid=28873087), [Fulu E](https://www.ncbi.nlm.nih.gov/pubmed/?term=Fulu%20E%5BAuthor%5D&cauthor=true&cauthor_uid=28873087), [Tabassam Naved R](https://www.ncbi.nlm.nih.gov/pubmed/?term=Tabassam%20Naved%20R%5BAuthor%5D&cauthor=true&cauthor_uid=28873087), [Chirwa E](https://www.ncbi.nlm.nih.gov/pubmed/?term=Chirwa%20E%5BAuthor%5D&cauthor=true&cauthor_uid=28873087), [Dunkle K](https://www.ncbi.nlm.nih.gov/pubmed/?term=Dunkle%20K%5BAuthor%5D&cauthor=true&cauthor_uid=28873087), [Haardörfer R](https://www.ncbi.nlm.nih.gov/pubmed/?term=Haard%C3%B6rfer%20R%5BAuthor%5D&cauthor=true&cauthor_uid=28873087), et al. Women's and men's reports of past-year prevalence of intimate partner violence and rape and women's risk factors for intimate partner violence: A multicountry cross-sectional study in Asia and the Pacific. [*PLoS Med*](https://www.ncbi.nlm.nih.gov/pubmed/?term=10.1371%2Fjournal.pmed.1002381)*,* 2017; 14:e1002381. |
| Lövestad S, Löve J, Vaez M, Krantz G. Prevalence of intimate partner violence and its association with symptoms of depression; a cross-sectional study based on a female population sample in Sweden. *BMC Public Health*, 2017; 17:335. |
| [Mohamadian F](https://www.ncbi.nlm.nih.gov/pubmed/?term=Mohamadian%20F%5BAuthor%5D&cauthor=true&cauthor_uid=27468345), [Hashemian A](https://www.ncbi.nlm.nih.gov/pubmed/?term=Hashemian%20A%5BAuthor%5D&cauthor=true&cauthor_uid=27468345), [Bagheri M](https://www.ncbi.nlm.nih.gov/pubmed/?term=Bagheri%20M%5BAuthor%5D&cauthor=true&cauthor_uid=27468345), [Direkvand-Moghadam A](https://www.ncbi.nlm.nih.gov/pubmed/?term=Direkvand-Moghadam%20A%5BAuthor%5D&cauthor=true&cauthor_uid=27468345). Prevalence and Risk Factors of Domestic Violence against Iranian Women: A Cross-Sectional Study. [*Korean J Fam Med*](https://www.ncbi.nlm.nih.gov/pubmed/?term=10.4082%2Fkjfm.2016.37.4.253), 2016; 37:253–8. |
| [de Barros ÉN](https://www.ncbi.nlm.nih.gov/pubmed/?term=de%20Barros%20%C3%89N%5BAuthor%5D&cauthor=true&cauthor_uid=26910166), [Silva MA](https://www.ncbi.nlm.nih.gov/pubmed/?term=Silva%20MA%5BAuthor%5D&cauthor=true&cauthor_uid=26910166), [Falbo Neto GH](https://www.ncbi.nlm.nih.gov/pubmed/?term=Falbo%20Neto%20GH%5BAuthor%5D&cauthor=true&cauthor_uid=26910166), [Lucena SG](https://www.ncbi.nlm.nih.gov/pubmed/?term=Lucena%20SG%5BAuthor%5D&cauthor=true&cauthor_uid=26910166), [Ponzo L](https://www.ncbi.nlm.nih.gov/pubmed/?term=Ponzo%20L%5BAuthor%5D&cauthor=true&cauthor_uid=26910166), [Pimentel AP](https://www.ncbi.nlm.nih.gov/pubmed/?term=Pimentel%20AP%5BAuthor%5D&cauthor=true&cauthor_uid=26910166). Prevalence and factors associated with intimate partner violence among women in Recife/Pernambuco, Brazil. [*Cien Saude Colet*](https://www.ncbi.nlm.nih.gov/pubmed/?term=10.1590%2F1413-81232015212.10672015), 2016; 21:591–8. |
| [Karakoç B](https://www.ncbi.nlm.nih.gov/pubmed/?term=Karako%C3%A7%20B%5BAuthor%5D&cauthor=true&cauthor_uid=28360734), [Gülseren L](https://www.ncbi.nlm.nih.gov/pubmed/?term=G%C3%BClseren%20L%5BAuthor%5D&cauthor=true&cauthor_uid=28360734), [Çam B](https://www.ncbi.nlm.nih.gov/pubmed/?term=%C3%87am%20B%5BAuthor%5D&cauthor=true&cauthor_uid=28360734), [Gülseren Ş](https://www.ncbi.nlm.nih.gov/pubmed/?term=G%C3%BClseren%20%C5%9E%5BAuthor%5D&cauthor=true&cauthor_uid=28360734), [Tenekeci N](https://www.ncbi.nlm.nih.gov/pubmed/?term=Tenekeci%20N%5BAuthor%5D&cauthor=true&cauthor_uid=28360734), [Mete L](https://www.ncbi.nlm.nih.gov/pubmed/?term=Mete%20L%5BAuthor%5D&cauthor=true&cauthor_uid=28360734). Prevalence of Intimate Partner Violence and Associated Factors. [*Noro Psikiyatr* *Ars*](https://www.ncbi.nlm.nih.gov/pubmed/?term=10.5152%2Fnpa.2015.7535), 2015; 52:324–330. |
| [Sugg N](https://www.ncbi.nlm.nih.gov/pubmed/?term=Sugg%20N%5BAuthor%5D&cauthor=true&cauthor_uid=25841604). Intimate partner violence: prevalence, health consequences, and intervention. [*Med Clin North Am*](https://www.ncbi.nlm.nih.gov/pubmed/?term=10.1016%2Fj.mcna.2015.01.012), 2015; 99:629–49. |
| [Breiding MJ](https://www.ncbi.nlm.nih.gov/pubmed/?term=Breiding%20MJ%5BAuthor%5D&cauthor=true&cauthor_uid=25188037), [Smith SG](https://www.ncbi.nlm.nih.gov/pubmed/?term=Smith%20SG%5BAuthor%5D&cauthor=true&cauthor_uid=25188037), [Basile KC](https://www.ncbi.nlm.nih.gov/pubmed/?term=Basile%20KC%5BAuthor%5D&cauthor=true&cauthor_uid=25188037), [Walters ML](https://www.ncbi.nlm.nih.gov/pubmed/?term=Walters%20ML%5BAuthor%5D&cauthor=true&cauthor_uid=25188037), [Chen J](https://www.ncbi.nlm.nih.gov/pubmed/?term=Chen%20J%5BAuthor%5D&cauthor=true&cauthor_uid=25188037), [Merrick MT](https://www.ncbi.nlm.nih.gov/pubmed/?term=Merrick%20MT%5BAuthor%5D&cauthor=true&cauthor_uid=25188037). [Prevalence and characteristics of sexual violence, stalking, and intimate partner violence victimization--national intimate partner and sexual violence survey, United States, 2011.](https://www.ncbi.nlm.nih.gov/pubmed/25188037) [*MMWR Surveill Summ*](https://www.ncbi.nlm.nih.gov/pubmed)*,* 2014; 63:1–18. |
| [Mishra A](https://www.ncbi.nlm.nih.gov/pubmed/?term=Mishra%20A%5BAuthor%5D&cauthor=true&cauthor_uid=24695623), [Patne S](https://www.ncbi.nlm.nih.gov/pubmed/?term=Patne%20S%5BAuthor%5D&cauthor=true&cauthor_uid=24695623), [Tiwari R](https://www.ncbi.nlm.nih.gov/pubmed/?term=Tiwari%20R%5BAuthor%5D&cauthor=true&cauthor_uid=24695623), [Srivastava DK](https://www.ncbi.nlm.nih.gov/pubmed/?term=Srivastava%20DK%5BAuthor%5D&cauthor=true&cauthor_uid=24695623), [Gour N](https://www.ncbi.nlm.nih.gov/pubmed/?term=Gour%20N%5BAuthor%5D&cauthor=true&cauthor_uid=24695623), [Bansal M](https://www.ncbi.nlm.nih.gov/pubmed/?term=Bansal%20M%5BAuthor%5D&cauthor=true&cauthor_uid=24695623). A Cross-sectional Study to Find out the Prevalence of Different Types of Domestic Violence in Gwalior City and to Identify the Various Risk and Protective Factors for Domestic Violence. [*Indian J Community Med*](https://www.ncbi.nlm.nih.gov/pubmed/?term=10.4103%2F0970-0218.126348), 2014; 39:21–5. |
| [Yari A](https://www.ncbi.nlm.nih.gov/pubmed/?term=Yari%20A%5BAuthor%5D&cauthor=true&cauthor_uid=24971119), [Nouri R](https://www.ncbi.nlm.nih.gov/pubmed/?term=Nouri%20R%5BAuthor%5D&cauthor=true&cauthor_uid=24971119), [Rashidian H](https://www.ncbi.nlm.nih.gov/pubmed/?term=Rashidian%20H%5BAuthor%5D&cauthor=true&cauthor_uid=24971119), [Nadrian H](https://www.ncbi.nlm.nih.gov/pubmed/?term=Nadrian%20H%5BAuthor%5D&cauthor=true&cauthor_uid=24971119). [Prevalence and determinants of sexual intimate partner violence against women in the city of marivan, iran.](https://www.ncbi.nlm.nih.gov/pubmed/24971119) [*J Family Reprod Health*](https://www.ncbi.nlm.nih.gov/pubmed), 2013; 7:157–63. |
| [Fulu E](https://www.ncbi.nlm.nih.gov/pubmed/?term=Fulu%20E%5BAuthor%5D&cauthor=true&cauthor_uid=25104345), [Jewkes R](https://www.ncbi.nlm.nih.gov/pubmed/?term=Jewkes%20R%5BAuthor%5D&cauthor=true&cauthor_uid=25104345), [Roselli T](https://www.ncbi.nlm.nih.gov/pubmed/?term=Roselli%20T%5BAuthor%5D&cauthor=true&cauthor_uid=25104345), [Garcia-Moreno C](https://www.ncbi.nlm.nih.gov/pubmed/?term=Garcia-Moreno%20C%5BAuthor%5D&cauthor=true&cauthor_uid=25104345); [UN Multi-country Cross-sectional Study on Men and Violence research team](https://www.ncbi.nlm.nih.gov/pubmed/?term=UN%20Multi-country%20Cross-sectional%20Study%20on%20Men%20and%20Violence%20research%20team%5BCorporate%20Author%5D). Prevalence of and factors associated with male perpetration of intimate partner violence: findings from the UN Multi-country Cross-sectional Study on Men and Violence in Asia and the Pacific. [*Lancet Glob Health*](https://www.ncbi.nlm.nih.gov/pubmed/25104345), 2013; 1:e187-207. |
| [Devries KM](https://www.ncbi.nlm.nih.gov/pubmed/?term=Devries%20KM%5BAuthor%5D&cauthor=true&cauthor_uid=23788730), [Mak JY](https://www.ncbi.nlm.nih.gov/pubmed/?term=Mak%20JY%5BAuthor%5D&cauthor=true&cauthor_uid=23788730), [García-Moreno C](https://www.ncbi.nlm.nih.gov/pubmed/?term=Garc%C3%ADa-Moreno%20C%5BAuthor%5D&cauthor=true&cauthor_uid=23788730), [Petzold M](https://www.ncbi.nlm.nih.gov/pubmed/?term=Petzold%20M%5BAuthor%5D&cauthor=true&cauthor_uid=23788730), [Child JC](https://www.ncbi.nlm.nih.gov/pubmed/?term=Child%20JC%5BAuthor%5D&cauthor=true&cauthor_uid=23788730), [Falder G](https://www.ncbi.nlm.nih.gov/pubmed/?term=Falder%20G%5BAuthor%5D&cauthor=true&cauthor_uid=23788730), [et](https://www.ncbi.nlm.nih.gov/pubmed/?term=Watts%20CH%5BAuthor%5D&cauthor=true&cauthor_uid=23788730) al. Global health. The global prevalence of intimate partner violence against women. *Science*, 2013; 340:1527–8. |
| [Meekers D](https://www.ncbi.nlm.nih.gov/pubmed/?term=Meekers%20D%5BAuthor%5D&cauthor=true&cauthor_uid=23534436), [Pallin SC](https://www.ncbi.nlm.nih.gov/pubmed/?term=Pallin%20SC%5BAuthor%5D&cauthor=true&cauthor_uid=23534436), [Hutchinson P](https://www.ncbi.nlm.nih.gov/pubmed/?term=Hutchinson%20P%5BAuthor%5D&cauthor=true&cauthor_uid=23534436). Prevalence and correlates of physical, psychological, and sexual intimate partner violence in Bolivia. [*Glob Public Health*](https://www.ncbi.nlm.nih.gov/pubmed/?term=10.1080%2F17441692.2013.776093), 2013; 8:588–606. |
| [Ismayilova L](https://www.ncbi.nlm.nih.gov/pubmed/?term=Ismayilova%20L%5BAuthor%5D&cauthor=true&cauthor_uid=23508086), [El-Bassel N](https://www.ncbi.nlm.nih.gov/pubmed/?term=El-Bassel%20N%5BAuthor%5D&cauthor=true&cauthor_uid=23508086). Prevalence and correlates of intimate partner violence by type and severity: population-based studies in Azerbaijan, Moldova, and Ukraine. [*J Interpers Violence*](https://www.ncbi.nlm.nih.gov/pubmed/?term=10.1177%2F0886260513479026), 2013; 28:2521–56. |
| [Barrett BJ](https://www.ncbi.nlm.nih.gov/pubmed/?term=Barrett%20BJ%5BAuthor%5D&cauthor=true&cauthor_uid=23081876), [Habibov N](https://www.ncbi.nlm.nih.gov/pubmed/?term=Habibov%20N%5BAuthor%5D&cauthor=true&cauthor_uid=23081876), [Chernyak E](https://www.ncbi.nlm.nih.gov/pubmed/?term=Chernyak%20E%5BAuthor%5D&cauthor=true&cauthor_uid=23081876). Factors affecting prevalence and extent of intimate partner violence in Ukraine: evidence from a nationally representative survey. [*Violence Against Women*](https://www.ncbi.nlm.nih.gov/pubmed/?term=10.1177%2F1077801212464387), 2012; 18:1147–76. |
| [Dalal K](https://www.ncbi.nlm.nih.gov/pubmed/?term=Dalal%20K%5BAuthor%5D&cauthor=true&cauthor_uid=21118856), [Lindqvist K](https://www.ncbi.nlm.nih.gov/pubmed/?term=Lindqvist%20K%5BAuthor%5D&cauthor=true&cauthor_uid=21118856). A national study of the prevalence and correlates of domestic violence among women in India. [*Asia Pac J Public Health*](https://www.ncbi.nlm.nih.gov/pubmed/?term=10.1177%2F1010539510384499), 2012; 24:265–77. |
| [Abeya SG](https://www.ncbi.nlm.nih.gov/pubmed/?term=Abeya%20SG%5BAuthor%5D&cauthor=true&cauthor_uid=22151213), [Afework MF](https://www.ncbi.nlm.nih.gov/pubmed/?term=Afework%20MF%5BAuthor%5D&cauthor=true&cauthor_uid=22151213), [Yalew AW](https://www.ncbi.nlm.nih.gov/pubmed/?term=Yalew%20AW%5BAuthor%5D&cauthor=true&cauthor_uid=22151213). Intimate partner violence against women in western Ethiopia: prevalence, patterns, and associated factors. [*BMC Public Health*](https://www.ncbi.nlm.nih.gov/pubmed/?term=10.1186%2F1471-2458-11-913), 2011; 11:913. |
| [Jayasuriya V](https://www.ncbi.nlm.nih.gov/pubmed/?term=Jayasuriya%20V%5BAuthor%5D&cauthor=true&cauthor_uid=21890530), [Wijewardena K](https://www.ncbi.nlm.nih.gov/pubmed/?term=Wijewardena%20K%5BAuthor%5D&cauthor=true&cauthor_uid=21890530), [Axemo P](https://www.ncbi.nlm.nih.gov/pubmed/?term=Axemo%20P%5BAuthor%5D&cauthor=true&cauthor_uid=21890530). Intimate partner violence against women in the capital province of Sri Lanka: prevalence, risk factors, and help seeking. [*Violence Against Women*](https://www.ncbi.nlm.nih.gov/pubmed/?term=10.1177%2F1077801211417151), 2011; 17:1086–102. |
| [Garcia-Moreno C](https://www.ncbi.nlm.nih.gov/pubmed/?term=Garcia-Moreno%20C%5BAuthor%5D&cauthor=true&cauthor_uid=17027732), [Jansen HA](https://www.ncbi.nlm.nih.gov/pubmed/?term=Jansen%20HA%5BAuthor%5D&cauthor=true&cauthor_uid=17027732), [Ellsberg M](https://www.ncbi.nlm.nih.gov/pubmed/?term=Ellsberg%20M%5BAuthor%5D&cauthor=true&cauthor_uid=17027732), [Heise L](https://www.ncbi.nlm.nih.gov/pubmed/?term=Heise%20L%5BAuthor%5D&cauthor=true&cauthor_uid=17027732), [Watts CH](https://www.ncbi.nlm.nih.gov/pubmed/?term=Watts%20CH%5BAuthor%5D&cauthor=true&cauthor_uid=17027732); [WHO Multi-country Study on Women's Health and Domestic Violence against Women Study Team](https://www.ncbi.nlm.nih.gov/pubmed/?term=WHO%20Multi-country%20Study%20on%20Women's%20Health%20and%20Domestic%20Violence%20against%20Women%20Study%20Team%5BCorporate%20Author%5D). [Prevalence of intimate partner violence: findings from the WHO multi-country study on women's health and domestic violence.](https://www.ncbi.nlm.nih.gov/pubmed/17027732) [*Lancet*](https://www.ncbi.nlm.nih.gov/pubmed)*,* 2006; 368:1260–9. |
| [Thompson RS](https://www.ncbi.nlm.nih.gov/pubmed/?term=Thompson%20RS%5BAuthor%5D&cauthor=true&cauthor_uid=16704937), [Bonomi AE](https://www.ncbi.nlm.nih.gov/pubmed/?term=Bonomi%20AE%5BAuthor%5D&cauthor=true&cauthor_uid=16704937), [Anderson M](https://www.ncbi.nlm.nih.gov/pubmed/?term=Anderson%20M%5BAuthor%5D&cauthor=true&cauthor_uid=16704937), [Reid RJ](https://www.ncbi.nlm.nih.gov/pubmed/?term=Reid%20RJ%5BAuthor%5D&cauthor=true&cauthor_uid=16704937), [Dimer JA](https://www.ncbi.nlm.nih.gov/pubmed/?term=Dimer%20JA%5BAuthor%5D&cauthor=true&cauthor_uid=16704937), [Carrell D](https://www.ncbi.nlm.nih.gov/pubmed/?term=Carrell%20D%5BAuthor%5D&cauthor=true&cauthor_uid=16704937), et al. [Intimate partner violence: prevalence, types, and chronicity in adult women.](https://www.ncbi.nlm.nih.gov/pubmed/16704937) [*Am J Prev Med*](https://www.ncbi.nlm.nih.gov/pubmed), 2006; 30:447–57. |
| [Faramarzi M](https://www.ncbi.nlm.nih.gov/pubmed/?term=Faramarzi%20M%5BAuthor%5D&cauthor=true&cauthor_uid=16761656), [Esmailzadeh S](https://www.ncbi.nlm.nih.gov/pubmed/?term=Esmailzadeh%20S%5BAuthor%5D&cauthor=true&cauthor_uid=16761656), [Mosavi S](https://www.ncbi.nlm.nih.gov/pubmed/?term=Mosavi%20S%5BAuthor%5D&cauthor=true&cauthor_uid=16761656). [Prevalence and determinants of intimate partner violence in Babol City, Islamic Republic of Iran.](https://www.ncbi.nlm.nih.gov/pubmed/16761656) [*East Mediterr Health J*](https://www.ncbi.nlm.nih.gov/pubmed), 2005; 11:870–9. |
| [Xu X](https://www.ncbi.nlm.nih.gov/pubmed/?term=Xu%20X%5BAuthor%5D&cauthor=true&cauthor_uid=15623864), [Zhu F](https://www.ncbi.nlm.nih.gov/pubmed/?term=Zhu%20F%5BAuthor%5D&cauthor=true&cauthor_uid=15623864), [O'Campo P](https://www.ncbi.nlm.nih.gov/pubmed/?term=O'Campo%20P%5BAuthor%5D&cauthor=true&cauthor_uid=15623864), [Koenig MA](https://www.ncbi.nlm.nih.gov/pubmed/?term=Koenig%20MA%5BAuthor%5D&cauthor=true&cauthor_uid=15623864), [Mock V](https://www.ncbi.nlm.nih.gov/pubmed/?term=Mock%20V%5BAuthor%5D&cauthor=true&cauthor_uid=15623864), [Campbell J](https://www.ncbi.nlm.nih.gov/pubmed/?term=Campbell%20J%5BAuthor%5D&cauthor=true&cauthor_uid=15623864). [Prevalence of and risk factors for intimate partner violence in China.](https://www.ncbi.nlm.nih.gov/pubmed/15623864) [*Am J Public Health*](https://www.ncbi.nlm.nih.gov/pubmed), 2005; 95:78–85. |
| [Parish WL](https://www.ncbi.nlm.nih.gov/pubmed/?term=Parish%20WL%5BAuthor%5D&cauthor=true&cauthor_uid=15590383), [Wang T](https://www.ncbi.nlm.nih.gov/pubmed/?term=Wang%20T%5BAuthor%5D&cauthor=true&cauthor_uid=15590383), [Laumann EO](https://www.ncbi.nlm.nih.gov/pubmed/?term=Laumann%20EO%5BAuthor%5D&cauthor=true&cauthor_uid=15590383), [Pan S](https://www.ncbi.nlm.nih.gov/pubmed/?term=Pan%20S%5BAuthor%5D&cauthor=true&cauthor_uid=15590383), [Luo Y](https://www.ncbi.nlm.nih.gov/pubmed/?term=Luo%20Y%5BAuthor%5D&cauthor=true&cauthor_uid=15590383). [Intimate partner violence in China: national prevalence, risk factors and associated health problems.](https://www.ncbi.nlm.nih.gov/pubmed/15590383) [*Int Fam Plan Perspect*](https://www.ncbi.nlm.nih.gov/pubmed), 2004 ; 30:174–81. |
| [Fanslow J](https://www.ncbi.nlm.nih.gov/pubmed/?term=Fanslow%20J%5BAuthor%5D&cauthor=true&cauthor_uid=15570342), [Robinson E](https://www.ncbi.nlm.nih.gov/pubmed/?term=Robinson%20E%5BAuthor%5D&cauthor=true&cauthor_uid=15570342). [Violence against women in New Zealand: prevalence and health consequences.](https://www.ncbi.nlm.nih.gov/pubmed/15570342) [*N Z Med J*](https://www.ncbi.nlm.nih.gov/pubmed), 2004; 117:1173. |
| [Clark JP](https://www.ncbi.nlm.nih.gov/pubmed/?term=Clark%20JP%5BAuthor%5D&cauthor=true&cauthor_uid=12583680), [Du Mont J](https://www.ncbi.nlm.nih.gov/pubmed/?term=Du%20Mont%20J%5BAuthor%5D&cauthor=true&cauthor_uid=12583680). [Intimate partner violence and health: a critique of Canadian prevalence studies.](https://www.ncbi.nlm.nih.gov/pubmed/12583680) [*Can J Public Health*](https://www.ncbi.nlm.nih.gov/pubmed), 2003; 94:52–8. |
| [Okemgbo CN](https://www.ncbi.nlm.nih.gov/pubmed/?term=Okemgbo%20CN%5BAuthor%5D&cauthor=true&cauthor_uid=12476722), [Omideyi AK](https://www.ncbi.nlm.nih.gov/pubmed/?term=Omideyi%20AK%5BAuthor%5D&cauthor=true&cauthor_uid=12476722), [Odimegwu CO](https://www.ncbi.nlm.nih.gov/pubmed/?term=Odimegwu%20CO%5BAuthor%5D&cauthor=true&cauthor_uid=12476722). [Prevalence, patterns and correlates of domestic violence in selected Igbo communities of Imo State, Nigeria.](https://www.ncbi.nlm.nih.gov/pubmed/12476722) [*Afr J Reprod Health*](https://www.ncbi.nlm.nih.gov/pubmed), 2002; 6:101–14. |
| [Wilt S](https://www.ncbi.nlm.nih.gov/pubmed/?term=Wilt%20S%5BAuthor%5D&cauthor=true&cauthor_uid=8683026), [Olson S](https://www.ncbi.nlm.nih.gov/pubmed/?term=Olson%20S%5BAuthor%5D&cauthor=true&cauthor_uid=8683026). [Prevalence of domestic violence in the United States.](https://www.ncbi.nlm.nih.gov/pubmed/8683026) [*J Am Med Womens Assoc* (1972)](https://www.ncbi.nlm.nih.gov/pubmed),1996; 51:77–82. |
| [Browne A](https://www.ncbi.nlm.nih.gov/pubmed/?term=Browne%20A%5BAuthor%5D&cauthor=true&cauthor_uid=8256881). [Violence against women by male partners. Prevalence, outcomes, and policy implications.](https://www.ncbi.nlm.nih.gov/pubmed/8256881) [*Am Psychol*](https://www.ncbi.nlm.nih.gov/pubmed), 1993; 48:1077–87. |

Appendix B. Definition of lifetime violence

| Did your current partner/previous partner: |
| --- |
|  |
| Physical |
| Push you or shove you? |
| Slap you? |
| Throw a hard object at you? |
| Grab you or pull your hair? |
| Beat you with a fist or a hard object, or kick you? |
| Burn you? |
| Try to suffocate you or strangle you? |
| Cut or stab you, or shoot at you? |
| Beat your head against something? |
|  |
| Sexual |
| Make you watch or look at pornographic material against wishes? |
| Force you into sexual intercourse by holding you down or hurting you in some way? |
| Attempt to force you into sexual intercourse by holding you down or hurt you in some way? |
| Make you take part in any form of sexual activity when you did not want to or you were unable to refuse? |
| Share intimate photos or videos of you, on the internet or by mobile phone? |
|  |
| Psychological |
| Unwelcome touch, hug or kiss you? |
| Inappropriately stare or leer at you that made you feel intimidated? |
| Make sexually suggestive comments or jokes that made you feel offended? |
| Send or show you sexually explicit pictures, photos or gifts that made you feel offended? |
| Inappropriate invite you to go out on dates? |
| Ask intrusive questions about your private life that made you feel offended? |
| Comment in an intrusive way your physical appearance that made you feel offended? |
| Send you unwanted sexually explicit emails or SMS messages that offended you? |
| Make inappropriate advances that offended you on social networking websites such as Facebook, or in internet chat rooms? |
| Indecently expose him to you? |
| Threat to hurt you physically? |
| Try to keep you from seeing friends? |
| Try to restrict contact with your family or birth relatives? |
| Insist on knowing where you are in a way that goes beyond general concern? |
| Get angry of you speak with another man? |
| Become suspicious that you are unfaithful? |
| Prevent from making decisions about family finances and from shopping independently? |
| Forbid you to work outside the home? |
| Forbid you to leave the house, take away car keys or lock you up? |
| Belittle or humiliate you in front of other people? |
| Belittle or humiliate you in private? |
| Things to scare or intimidate you on purpose for example by yelling and smashing things? |
| Threat to take children away? |
| Threat to hurt children? |
| Hurt children? |
| Threat to hurt someone you care about? |
| Send emails, text messages (SMS) or instant messages that are offensive or threatening? |
| Send letters or cards that are offensive or threatening? |
| Make offensive threatening or silent calls? |
| Poste offensive comments about you on the internet? |
| Loiter or wait you outside the home, workplace or school without a legitimate reason? |
| Deliberately follow you around? |
| Deliberately interfere with or damage your property? |
| Threat concerning the custody of the children when/after splitting or separating? |
| Threat to hurt children when/after splitting or separating? |
| Hurt children when/after splitting or separating? |
| Hurt you or threat to hurt you when visiting, picking or bringing back the children when/after splitting or separating? |
|  |
| Did you feel to have an unequal say with regard to the use of the household income? |

Appendix C. Definition of violence before the age of 15

| Before you were 15 years old, did any adult: |
| --- |
|  |
| Slap you or pull your hair so that it hurt you? |
| Hit you very hard so that it hurt you? |
| Kick you very hard so that it hurt you? |
| Beat you very hard with an object like a stick, cane or belt so that it hurt you? |
| Stab or cut you with something so that it hurt you? |
| Threaten to hurt you badly or kill you? |
| Expose their genitals to you when you did not want them to? |
| Make you pose naked in front of any person or in photographs, video or an internet webcam when you did not want to do this? |
| Touch your private parts – genitals or breasts – when you did not want them to? |
| Make you touch their private parts – genitals or breasts – when you did not want to do this? |
| Make you have sexual intercourse with them when you did not want to? |
|  |
| Before you were 15 years old, how often did any adult member of your family – who was 18 years or older – do any of the following to you: |
|  |
| Say to you that you were not loved? |
| Say that they wished you had never been born? |
| Threaten to abandon you or throw you out of the family home? |

Appendix D. Associations between physical IPV and ever-partnered women characteristics

|  | Physical IPV non-victim women | Physical IPV victim women | OR | | (95%CI) |  |  |
| --- | --- | --- | --- | --- | --- | --- | --- |
|  | N=12019 | (%) | | N=8075 | (%) |  |  |
| Age |  |  | |  |  |  |  |
| 18-29 | 2429 | (20·2) | | 1365 | (16·9) | Ref |  |
| 30-39 | 3053 | (25·4) | | 1702 | (21·1) | 0·99 | (0·85-1·16) |
| 40-49 | 2988 | (24·9) | | 1911 | (23·7) | 1·38 | (0·96-1·35) |
| 50-59 | 2044 | (17·0) | | 1528 | (18·9) | 1·33 | (1·14-1·56) |
| ≥ 60 | 1486 | (12·4) | | 1560 | (19·3) | 1·87 | (1·59-2·20) |
| Highest level of education |  |  | |  |  |  |  |
| Primary | 3062 | (25·5) | | 3214 | (39·8) | Ref |  |
| Secondary | 5772 | (48·0) | | 3414 | (42·3) | 0·56 | (0·50-0·64) |
| Tertiary | 3165 | (26·3) | | 1433 | (17·7) | 0·43 | (0·37-0·50) |
| Employment status |  |  | |  |  |  |  |
| Paid work | 7703 | (64·1) | | 4274 | (52·9) | Ref |  |
| Retired | 1101 | (9·2) | | 1187 | (14·7) | 1·94 | (1·67-2·27) |
| Home maker | 1374 | (11·4) | | 999 | (12·4) | 1·31 | (1·12-1·53) |
| Student, in training | 710 | (5·9) | | 400 | (5·0) | 1·01 | (0·78-1·31) |
| Unemployed | 852 | (7·1) | | 882 | (10·9) | 1·87 | (1·54-2·26) |
| Other | 260 | (2·2) | | 323 | (4·0) | 2·24 | (1·69-2·96) |
| Employment during past 12 months | 1232 | (10·2) | | 998 | (12·4) | 0·02 | (0·73-1·08) |
| Occupation type |  |  | |  |  |  |  |
| Employed | 5558 | (46·2) | | 3287 | (40·7) | Ref |  |
| Farmer or fisherwomen | 97 | (0·8) | | 202 | (2·5) | 3·54 | (2·33-5·39) |
| Professional | 600 | (5·0) | | 280 | (3·5) | 0·78 | (0·59-1·06) |
| Owner | 794 | (6·6) | | 481 | (6·0) | 1·02 | (0·81-1·30) |
| Manager | 1812 | (15·1) | | 926 | (11·5) | 0·86 | (0·72-1·03) |
| Supervisor | 81 | (0·7) | | 73 | (0·9) | 1·51 | (0·87-2·61) |
| Manual worker/servant | 2384 | (19·8) | | 2346 | (29·0) | 1·66 | (1·46-1·89) |
| Never done paid work | 625 | (5·2) | | 457 | (5·7) | 1·24 | (0·96-1·59) |
| Citizen | 11645 | (96·9) | | 7728 | (95·7) | 0·69 | (0·51-0·95) |
| At least one migrated parent | 1353 | (11·3) | | 1199 | (14·8) | 1·38 | (1·18-1·61) |
| Type of locality |  |  | |  |  |  |  |
| Urban | 8588 | (71·4) | | 6057 | (75·0) | Ref |  |
| Rural | 3400 | (28·3) | | 1985 | (24·6) | 1·13 | (0·93-1·37) |
| Married or in a civil partnership | 8649 | (72·0) | | 4001 | (49·5) | 0·38 | (0·35-0·43) |
| Cohabitation with a partner | 9940 | (82·7) | | 4495 | (55·7) | 0·27 | (0·24-0·31) |
| Children | 8690 | (72·3) | | 6280 | (77·8) | 1·34 | (1·17-1·52) |
| Number of people living in the household |  |  | |  |  |  |  |
| Under the age of 18 |  |  | |  |  |  |  |
| 0 | 5877 | (48·9) | | 4000 | (49·5) | Ref |  |
| 1 | 2597 | (21·6) | | 1843 | (22·8) | 1·04 | (0·92-1·19) |
| 2 or more | 3352 | (27·9) | | 2030 | (25·1) | 0·89 | (0·78-1·01) |
| Aged 18 and over |  |  | |  |  |  |  |
| 1 | 797 | (6·6) | | 1932 | (23·9) | Ref |  |
| 2 or more | 11148 | (92·8) | | 6058 | (75·0) | 0·22 | (0·19-0·26) |
| Last relationship |  |  | |  |  |  |  |
| No | 0 | (0·0) | | 866 | (10·7) |  |  |
| Separation | 8941 | (74·4) | | 4272 | (52·9) | NA |  |
| Divorce | 1300 | (10·8) | | 1941 | (24·0) | NA |  |
| Widowhood | 364 | (3·0) | | 521 | (6·4) | NA |  |
| Perceived healthiness |  |  | |  |  |  |  |
| Good | 9812 | (81·6) | | 4989 | (61·8) | Ref |  |
| Fair | 1783 | (14·8) | | 2188 | (27·1) | 2·41 | (2·13-2·74) |
| Bad | 446 | (3·5) | | 897 | (11·1) | 4·24 | (3·43-5·23) |
| Limitation | 1126 | (9·4) | | 1791 | (22·2) | 2·76 | (2·41-3·17) |
| Perceived disability | 295 | (2·5) | | 615 | (7·6) | 1·51 | (1·11-2·04) |
| Victim of violence before the age of 15 | 3999 | (33·3) | | 4498 | (55·7) | 2·55 | (2·32-2·80) |
| Victim of domestic violence before the age of 15 | 3161 | (26·3) | | 3745 | (46·4) | 2·45 | (2·19-2·75) |

*Source.* FRA Violence Against Women Survey dataset, 2012.

IPV=intimate partner violence. OR=relative risk reduction. CI=confidence interval. NA=not applicable.

When not specified, “No” is used as reference.

OR computed with binomial logistic regression models.

For each variable, the reference category is indicated as “Ref” (e.g. for age, the 18-29 age class is the category of reference for OR).

MD were considered as category.

Appendix E. Associations between sexual IPV and ever-partnered women characteristics

|  | Sexual IPV non-victim women | | Sexual IPV victim women | | OR | (95%CI) |
| --- | --- | --- | --- | --- | --- | --- |
|  | N=14260 | (%) | N=3404 | (%) |  |  |
| Age |  |  |  |  |  |  |
| 18-29 | 2965 | (20·8) | 482 | (14·2) | Ref |  |
| 30-39 | 3669 | (25·7) | 715 | (21·0) | 0·24 | (0·18-0·33) |
| 40-49 | 3549 | (24·9) | 861 | (25·3) | 0·19 | (0·14-0·25) |
| 50-59 | 2388 | (16·7) | 653 | (19·3) | 0·24 | (0·17-0·34) |
| ≥ 60 | 1670 | (11·7) | 691 | (20·3) | 0·33 | (0·24-0·46) |
| Highest level of education |  |  |  |  |  |  |
| Primary | 3737 | (26·2) | 1388 | (40·8) | Ref |  |
| Secondary | 6862 | (48·1) | 1376 | (40·4) | 0·81 | (0·68-0·96) |
| Tertiary | 3640 | (25·5) | 616 | (18·1) | 0·93 | (0·76-1·13) |
| Employment status |  |  |  |  |  |  |
| Paid work | 9043 | (63·4) | 1811 | (53·2) | Ref |  |
| Retired | 1253 | (8·8) | 501 | (14·7) | 2·00 | (1·64-2·43) |
| Home maker | 1639 | (11·5) | 426 | (12·5) | 1·30 | (1·03-1·63) |
| Student, in training | 880 | (6·2) | 137 | (4·0) | 0·78 | (0·54-1·13) |
| Unemployed | 1102 | (7·7) | 332 | (9·7) | 1·50 | (1·15-1·96) |
| Other | 324 | (2·3) | 193 | (5·7) | 2·97 | (2·08-4·26) |
| Employment during past 12 months | 1532 | (10·7) | 404 | (11·9) | 0·10 | (0·65-1·04) |
| Occupation type |  |  |  |  |  |  |
| Employed | 6624 | (46·5) | 1309 | (38·5) | Ref |  |
| Farmer or fisherwomen | 110 | (0·8) | 127 | (3·7) | 5·85 | (2·93-11·65) |
| Professional | 660 | (4·6) | 125 | (3·7) | 0·96 | (0·66-1·37) |
| Owner | 955 | (6·7) | 198 | (5·8) | 1·05 | (0·79-1·40) |
| Management | 2111 | (14·8) | 418 | (12·3) | 1·00 | (0·82-1·23) |
| Supervisor | 97 | (0·7) | 37 | (1·1) | 1·93 | (0·99-3·76) |
| Manual worker/servant | 2917 | (20·5) | 1004 | (29·5) | 1·74 | (1·46-2·08) |
| Never done paid work | 716 | (5·0) | 170 | (5·0) | 1·20 | (0·88-1·64) |
| Citizen | 13785 | (96·7) | 3262 | (95·8) | 0·77 | (0·49-1·23) |
| At least one migrated parent | 1677 | (11·8) | 521 | (15·3) | 1·37 | (1·11-1·68) |
| Type of locality |  |  |  |  |  |  |
| Urban | 10367 | (72·7) | 2514 | (73·9) | Ref |  |
| Rural | 3854 | (27·0) | 876 | (25·7) | 1·22 | (0·93-1·61) |
| Married or in a civil partnership | 10079 | (70·7) | 1595 | (46·9) | 0·37 | (0·31-0·43) |
| Cohabitation with a partner | 11682 | (81·9) | 1835 | (53·9) | 0·27 | (0·23-0·31) |
| Children | 10289 | (72·2) | 2708 | (79·6) | 1·50 | (1·24-1·80) |
| Number of people living in the household |  |  |  |  |  |  |
| Under the age of 18 |  |  |  |  |  |  |
| 0 | 6793 | (47·6) | 1719 | (50·5) | Ref |  |
| 1 | 3185 | (22·3) | 701 | (20·6) | 0·87 | (0·73-1·03) |
| 2 or more | 4002 | (28·1) | 932 | (27·4) | 0·92 | (0·77-1·10) |
| Aged 18 and over |  |  |  |  |  |  |
| 1 | 1048 | (7·3) | 855 | (25·1) | Ref |  |
| 2 or more | 13110 | (91·9) | 2519 | (74·0) | 0·24 | (0·20-0·28) |
| Last relationship |  |  |  |  |  |  |
| No | 0 | (0·00) | 281 | (8·3) |  |  |
| Separation | 10455 | (73·3) | 1800 | (52·9) | NA |  |
| Divorce | 1876 | (13·2) | 895 | (26·3) | NA |  |
| Widowhood | 435 | (3·1) | 244 | (7·2) | NA |  |
| Perceived healthiness |  |  |  |  |  |  |
| Good | 11461 | (80·4) | 1995 | (58·6) | Ref |  |
| Fair | 2209 | (15·5) | 1005 | (29·5) | 1·57 | (1·30-1·89) |
| Bad | 582 | (4·1) | 403 | (11·8) | 3·03 | (1·95-4·70) |
| Limitation | 1528 | (10·7) | 846 | (24·8) | 2·76 | (2·30-3·32) |
| Perceived disability | 415 | (2·9) | 288 | (8·5) | 1·45 | (1·04-2·01) |
| Victim of violence before the age of 15 | 5237 | (36·7) | 2093 | (61·5) | 2·80 | (2·7-3·17) |
| Victim of domestic violence before the age of 15 | 4187 | (29·4) | 1762 | (51·8) | 2·62 | (2·28-3·01) |

*Source.* FRA Violence Against Women Survey dataset, 2012.

IPV=intimate partner violence. NA=not applicable. OR=odd-ratio. CI=confidence interval. NA=not applicable

When not specified, “No” is used as reference.

OR computed with binomial logistic regression models.

For each variable, the reference category is indicated as “Ref” (e.g. for age, the 18-29 age class is the category of reference for OR).

MD were considered as category.

Appendix F. Associations between psychological IPV and ever-partnered women characteristics

|  | Psychological IPV non-victim women | | Psychological IPV victim women | | OR | (95%CI) |
| --- | --- | --- | --- | --- | --- | --- |
|  | N=2371 | (%) | N=20429 | (%) |  |  |
| Age |  |  |  |  |  |  |
| 18-29 | 144 | (6·1) | 4346 | (21·3) | Ref |  |
| 30-39 | 609 | (25·7) | 4440 | (21·7) | 0·24 | (0·18-0·33) |
| 40-49 | 750 | (31·6) | 4197 | (20·5) | 0·19 | (0·13-0·26) |
| 50-59 | 488 | (20·6) | 3603 | (17·6) | 0·24 | (0·18-0·34) |
| ≥ 60 | 380 | (16·0) | 3806 | (18·6) | 0·33 | (0·23-0·48) |
| Highest level of education |  |  |  |  |  |  |
| Primary | 718 | (30·3) | 6923 | (33·9) | Ref |  |
| Secondary | 1166 | (49·2) | 9107 | (44·6) | 0·81 | (0·68-0·96) |
| Tertiary | 484 | (20·4) | 4318 | (21·1) | 0·93 | (0·74-1·16) |
| Employment status |  |  |  |  |  |  |
| Paid work | 1492 | (62·9) | 11265 | (55·1) | Ref |  |
| Retired | 306 | (12·9) | 2971 | (14·5) | 1·29 | (1·07-1·55) |
| Home maker | 359 | (15·1) | 2278 | (11·2) | 0·84 | (0·67-1·06) |
| Student, in training | 7 | (0·3) | 1339 | (6·6) | 24·15 | (8·06-72·40) |
| Unemployed | 179 | (7·5) | 1875 | (9·2) | 1·39 | (1·03-1·87) |
| Other | 24 | (1·0) | 659 | (3·2) | 3·61 | (1·03-1·87) |
| Employment during past 12 months | 141 | (5·9) | 2414 | (11·8) | 1.90 | (1·38-2·62) |
| Occupation type |  |  |  |  |  |  |
| Employed | 1088 | (45·9) | 8614 | (42·2) | Ref |  |
| Farmer or fisherwomen | 44 | (1·8) | 356 | (1·7) | 1·03 | (0·63-1·67) |
| Professional | 83 | (3·5) | 837 | (4·1) | 1·28 | (0·84-1·95) |
| Owner | 218 | (9·2) | 1174 | (5·7) | 0·68 | (0·51-0·91) |
| Management | 323 | (13·6) | 2541 | (12·4) | 0·99 | (0·80-1·22) |
| Supervisor | 12 | (0·5) | 165 | (0·8) | 1·70 | (1·03-2·80) |
| Manual worker/servant | 461 | (19·5) | 5252 | (25·7) | 1·44 | (1·20-1·73) |
| Never done paid work | 120 | (5·0) | 1382 | (6·8) | 1·46 | (1·10-1·93) |
| Citizen | 2299 | (97·0) | 19616 | (96·0) | 0·75 | (0·49-1·14) |
| At least one migrated parent | 225 | (9·5) | 2772 | (13·6) | 1·52 | (1·21-1·90) |
| Type of locality |  |  |  |  |  |  |
| Urban | 1619 | (68·3) | 14782 | (72·4) | Ref |  |
| Rural | 748 | (31·5) | 5556 | (27·2) | 0·53 | (0·38-0·73) |
| Married or in a civil partnership | 2082 | (87·8) | 11036 | (54·0) | 0·16 | (0·13-0·21) |
| Cohabitation with a partner | 2228 | (94·0) | 12658 | (61·8) | 0·11 | (0·08-0·15) |
| Children | 2371 | (100·0) | 13615 | (66·6) | - | - |
| Number of people living in the household |  |  |  |  |  |  |
| Under the age of 18 |  |  |  |  |  |  |
| 0 | 941 | (39·7) | 11413 | (55·9) | Ref |  |
| 1 | 631 | (26·6) | 4030 | (19·7) | 0·53 | (0·44-0·63) |
| 2 or more | 774 | (32·6) | 4509 | (22·1) | 0·48 | (0·40-0·58) |
| Aged 18 and over |  |  |  |  |  |  |
| 1 | 97 | (4·1) | 3838 | (18·8) | Ref |  |
| 2 or more | 2253 | (95·0) | 16404 | (80·3) | 0·18 | (0·13-0·26) |
| Last relationship |  |  |  |  |  |  |
| No | 0 | (0·00) | 4091 | (20·0) |  |  |
| Separation | 1501 | (63·3) | 10503 | (51·4) | NA |  |
| Divorce | 321 | (13·5) | 3290 | (16·1) | NA |  |
| Widowhood | 110 | (4·6) | 1211 | (5·9) | NA |  |
| Perceived healthiness |  |  |  |  |  |  |
| Good | 1903 | (80·3) | 14199 | (69·5) | Ref |  |
| Fair | 392 | (16·6) | 4584 | (22·4) | 1·57 | (1·30-1·89) |
| Bad | 72 | (3·0) | 1630 | (8·0) | 3·03 | (1·89-4·85) |
| Limitation | 190 | (8·0) | 3330 | (16·3) | 2·25 | (1·67-3·01) |
| MD (N=5) |  |  |  |  |  |  |
| Perceived disability | 41 | (1·7) | 1118 | (5·5) | 1·83 | (1·12-2·98) |
| MD (N=2151) |  |  |  |  |  |  |
| Victim of violence before the age of 15 | 495 | (20·9) | 9309 | (45·6) | 3·24 | (2·74-3·84) |
| Victim of domestic violence before the age of 15 | 389 | (16·4) | 7596 | (37·2) | 3·09 | (2·52-3·79) |

*Source.* FRA Violence Against Women Survey dataset, 2012.

IPV=intimate partner violence. OR=relative risk reduction. CI=confidence interval. NA=not applicable.

When not specified, “No” is used as reference.

OR computed with binomial logistic regression models.

For each variable, the reference category is indicated as “Ref” (e.g. for age, the 18-29 age class is the category of reference for OR).

MD were considered as category.

Appendix G. Associations between physical IPV and characteristics of current partners of IPV victims

|  | Physical IPV non-perpetrator current partners | | Physical IPV perpetrator current partners | | OR | (95%CI) |
| --- | --- | --- | --- | --- | --- | --- |
|  | N=28104 | (%) | N=2178 | (%) |  |  |
| Age |  |  |  |  |  |  |
| 18-29 | 3762 | (13·4) | 195 | (9·0) | Ref |  |
| 30-39 | 5648 | (20·1) | 388 | (17·8) | 1·32 | (0·97-1·80) |
| 40-49 | 6250 | (22·2) | 538 | (24·7) | 1·66 | (1·19-2·31) |
| 50-59 | 5716 | (20·3) | 471 | (21·6) | 1·59 | (1·16-2·17) |
| ≥ 60 | 6595 | (23·5) | 578 | (26·5) | 1·69 | (1·25-2·28) |
| Highest level of education |  |  |  |  |  |  |
| Primary | 2019 | (7·2) | 326 | (14·9) | Ref |  |
| Secondary | 20090 | (71·5) | 1509 | (69·3) | 0·47 | (0·38-0·57) |
| Tertiary | 5615 | (20·0) | 320 | (14·7) | 0·35 | (0·27-0·45) |
| Employment status |  |  |  |  |  |  |
| Paid work | 19970 | (71·1) | 1433 | (65·8) | Ref |  |
| Retired | 5172·4 | (18·4) | 446 | (2·5) | 1·20 | (1·01-1·42) |
| Home maker | 112 | (0·4) | 33 | (1·5) | 4·12 | (2·16-7·83) |
| Student, in training | 803 | (2·9) | 41 | (1·9) | 7·11 | (3·57-1·41) |
| Unemployed | 1267 | (4·5) | 146 | (6·7) | 1·60 | (1·19-2·15) |
| Other | 595 | (2·1) | 71 | (3·3) | 1·67 | (1·09-2·57) |
| Occupation type |  |  |  |  |  |  |
| Employed | 6604 | (23·5) | 462 | (21·2) | Ref |  |
| Farmer or fisherwomen | 760 | (2·7) | 138 | (6·3) | 2·59 | (1·75-3·84) |
| Professional | 1256 | (4·5) | 99 | (4·5) | 1·12 | (0·75-1·69) |
| Owner | 2894 | (10·3) | 246 | (11·3) | 1·22 | (0·86-1·72) |
| Management | 4706 | (16·7) | 330 | (15·2) | 1·00 | (0·76-1·32) |
| Supervisor | 629 | (2·2) | 26 | (12) | 0·59 | (0·30-1·16) |
| Manual worker/servant | 10300 | (36·6) | 840 | (28·6) | 1·17 | (0·94-1·45) |
| Never done paid work | 623 | (2·2) | 26 | (1·2) | 0·60 | (0·29-1·24) |
| Drunkenness |  |  |  |  |  |  |
| Less than a couple of times a month | 26984 | (96·0) | 1512 | (69·4) | Ref |  |
| Once or twice a week | 525 | (1·9) | 504 | (23·1) | 3·95 | (2·91-5·36) |
| Every day | 124 | (0·4) | 162 | (7·5) | 12·81 | (8·23-16·95) |
| Violence outside the family | 1718 | (6·1) | 565 | (24·2) | 4·37 | (4·07-6·12) |
| Earning |  |  |  |  |  |  |
| Roughly the same than woman | 5463 | (19·4) | 383 | (17·6) | Ref |  |
| More than woman | 17740 | (63·1) | 1384 | (63·5) | 1·11 | (0·88-1·41) |
| Less than woman | 3151 | (11·2) | 304 | (13·9) | 1·37 | (0·97-1·94) |
| Length of relation with woman |  |  |  |  |  |  |
| < 1 year | 1004 | (3·6) | 68 | (3·1) | Ref |  |
| 1 - 10 years | 8922 | (31·7) | 557 | (25·6) | 0·92 | (0·50-1·72) |
| 11 - 20 years | 5627 | (20·0) | 477 | (21·9) | 1·26 | (0·67-2·34) |
| 20 - 30 years | 4819 | (17·1) | 425 | (19·5) | 1·31 | (0·70-4·44) |
| > 30 years | 7130 | (25·4) | 593 | (27·2) | 1·23 | (0·66-2·44) |

*Source.* FRA Violence Against Women Survey dataset, 2012.

IPV=intimate partner violence. OR=odd-ratio. CI=confidence interval. MD=missing data.

When not specified, “No” is used as reference.

OR computed with binomial logistic regression models.

For each variable, the reference category is indicated as “Ref” (e.g. for age, the 18-29 age class is the category of reference for OR).

MD were considered as category.

Appendix H. Associations between sexual IPV and characteristics of current partners of IPV victims

|  | Sexual IPV non-perpetrator current partners | | Sexual IPV perpetrator current partners | | OR | (95%CI) |
| --- | --- | --- | --- | --- | --- | --- |
|  | N=28699 | (%) | N=717 | (%) |  |  |
| Age |  |  |  |  |  |  |
| 18-29 | 3901 | (13·6) | 25 | (6·3) | Ref |  |
| 30-39 | 5850 | (20·4) | 126 | (17·3) | 3·32 | (1·86-5·94) |
| 40-49 | 6515 | (22·7) | 170 | (22·4) | 4·03 | (2·26-7·20) |
| 50-59 | 5881 | (20·5) | 149 | (20·8) | 3·92 | (2·21-6·94) |
| ≥ 60 | 6414 | (22·4) | 238 | (33·1) | 5·72 | (3·28-9·99) |
| Highest level of education |  |  |  |  |  |  |
| Primary | 2027 | (7·1) | 152 | (21·2) | Ref |  |
| Secondary | 20481 | (71·4) | 477 | (66·5) | 0·84 | (0·70-1·00) |
| Tertiary | 5793 | (20·2) | 77 | (10·7) | 0·82 | (0·69-0·99) |
| Employment status |  |  |  |  |  |  |
| Paid work | 20625 | (71·9) | 467 | (65·2) | Ref |  |
| Retired | 5002 | (17·4) | 140 | (19·6) | 1·24 | (0·86-1·79) |
| Home maker | 110 | (0·4) | 15 | (2·0) | 5·88 | (1·57-22·0) |
| Student, in training | 839 | (2·9) | 4 | (0·6) | 0·22 | (0·06-0·83) |
| Unemployed | 1320 | (4·6) | 46 | (6·4) | 1·53 | (0·94-2·48) |
| Other | 613 | (2·1) | 35 | (4·9) | 2·52 | (1·32-4·82) |
| Occupation type |  |  |  |  |  |  |
| Employed | 6797 | (23·7) | 103 | (14·4) | Ref |  |
| Farmer or fisherwomen | 741 | (2·6) | 73 | (10·1) | 6·47 | (3·01-13·89) |
| Professional | 1309 | (4·6) | 46 | (6·3) | 2·29 | (1·11-4·71) |
| Owner | 2955 | (10·3) | 118 | (16·5) | 2·64 | (1·40-4·99) |
| Management | 4867 | (17·0) | 80 | (11·1) | 1·08 | (0·66-1·77) |
| Supervisor | 619 | (2·2) | 21 | (2·9) | 2·23 | (0·96-5·16) |
| Manual worker/servant | 10447 | (36·4) | 257 | (35·9) | 1·62 | (1·08-2·44) |
| Never done paid work | 635 | (2·2) | 7 | (1·0) | 0·72 | (0·05-11·48) |
| Earning |  |  |  |  |  |  |
| Roughly the same than woman | 5572 | (19·4) | 139 | (19·3) | Ref |  |
| More than woman | 18115 | (63·1) | 411 | (57·3) | 0·91 | (0·59-1·40) |
| Less than woman | 3251 | (11·3) | 110 | (15·3) | 1·36 | (0·72-2·57) |
| Length of relation with woman |  |  |  |  |  |  |
| < 1 year | 1022 | (3·6) | 37 | (5·2) | Ref |  |
| 1 - 10 years | 9174 | (32·0) | 219 | (30·5) | 0·66 | (0·29-1·50) |
| 11 - 20 years | 5852 | (20·4) | 144 | (20·0) | 0·68 | (0·28-1·63) |
| 21 - 30 years | 5026 | (17·5) | 90 | (12·5) | 0·49 | (0·21-1·16) |
| > 30 years | 7009 | (24·4) | 190 | (26·5) | 0·75 | (0·32-1·78) |
| Drunkenness |  |  |  |  |  |  |
| Less than a couple of times a month | 27449 | (95·6) | 601 | (83·8) | Ref |  |
| Once or twice a week | 603 | (2·1) | 49 | (6·8) | 3·69 | (2·31-5·90) |
| Every day | 171 | (0·6) | 48 | (6·7) | 12·78 | (7·50-21·79) |
| Violence outside the family | 1960 | (6·8) | 217 | (30·3) | 4·66 | (4·60-9·16) |

*Source.* FRA Violence Against Women Survey dataset, 2012.

IPV=intimate partner violence. OR=odd-ratio. CI=confidence interval. MD=missing data.

When not specified, “No” is used as reference.

OR computed with binomial logistic regression models.

For each variable, the reference category is indicated as “Ref” (e.g. for age, the 18-29 age class is the category of reference for OR).

MD were considered as category.

Appendix I. Associations between psychological IPV and characteristics of current partners of IPV victims

|  | Psychological IPV non-perpetrator current partners | | Psychological IPV perpetrator current partners | | OR | (95%CI) |
| --- | --- | --- | --- | --- | --- | --- |
|  | N=14510 | (%) | N=10720 | (%) |  |  |
| Age |  |  |  |  |  |  |
| 18-29 | 619 | (4·3) | 1787 | (16·7) | Ref |  |
| 30-39 | 2977 | (20·5) | 2368 | (22·1) | 0·28 | (0·23-0·33) |
| 40-49 | 3906 | (26·9) | 2120 | (19·8) | 0·19 | (0·15-0·23) |
| 50-59 | 3508 | (24·2) | 1930 | (18·0) | 0·19 | (0·16-0·23) |
| ≥ 60 | 3474 | (23·9) | 2375 | (22·2) | 0·24 | (0·19-0·30) |
| Highest level of education |  |  |  |  |  |  |
| Primary | 1020 | (7·0) | 878 | (8·2) | Ref |  |
| Secondary | 10454 | (72·0) | 7554 | (70·5) | 0·84 | (0·71-0·99) |
| Tertiary | 2904 | (20·0) | 2059 | (19·2) | 0·82 | (0·69-0·99) |
| Employment status |  |  |  |  |  |  |
| Paid work | 10858 | (74·8) | 7465 | (69·6) | Ref |  |
| Retired | 2612 | (18·0) | 1909 | (17·8) | 1·06 | (0·94-1·20) |
| Home maker | 53 | (0·4) | 76 | (0·7) | 2·08 | (1·18-3·66) |
| Student, in training | 37 | (0·3) | 319 | (3·0) | 12·56 | (5·50-28·69) |
| Unemployed | 572 | (3·9) | 562 | (5·2) | 1·43 | (1·16-1·76) |
| Other | 334 | (2·3) | 244 | (2·3) | 1·06 | (0·76-1·50) |
| Occupation type |  |  |  |  |  |  |
| Employed | 3452 | (23·8) | 2442 | (22·8) | Ref |  |
| Farmer or fisherwomen | 427 | (2·9) | 318 | (3·0) | 1·05 | (0·81-1·38) |
| Professional | 635 | (4·4) | 509 | (4·7) | 1·13 | (0·94-1·37) |
| Owner | 1641 | (11·3) | 1085 | (10·1) | 0·93 | (0·79-1·11) |
| Management | 2602 | (17·9) | 1728 | (16·1) | 0·94 | (0·82-1·07) |
| Supervisor | 343 | (2·4) | 207 | (1·9) | 0·85 | (0·62-1·18) |
| Manual worker/servant | 5268 | (36·3) | 3998 | (37·3) | 1·07 | (0·96-1·20) |
| Never done paid work | 61 | (0·4) | 224 | (2·1) | 5·23 | (3·30-8·27) |
| Earning |  |  |  |  |  |  |
| Roughly the same than woman | 2919 | (20·1) | 2044 | (19·1) | Ref |  |
| More than woman | 9466 | (65·2) | 6578 | (61·4) | 0·99 | (0·88-1·12) |
| Less than woman | 1626 | (11·2) | 1409 | (13·1) | 1·24 | (1·04-1·47) |
| Length of the relation with woman |  |  |  |  |  |  |
| < 1 year | 151 | (1·0) | 326 | (3·0) | Ref |  |
| 1 - 10 years | 3213 | (22·1) | 4367 | (40·7) | 0·63 | (0·17-0·34) |
| 11 - 20 years | 3689 | (25·4) | 1909 | (17·8) | 0·24 | (0·16-0·33) |
| 21 – 30 years | 3107 | (21·4) | 1537 | (14·3) | 0·23 | (0·17-0·36) |
| > 30 years | 4110 | (28·3) | 2229 | (20·8) |  |  |
| Drunkenness |  |  |  |  |  |  |
| Less than a couple of times a month | 14042 | (96·8) | 9958 | (92·9) | Ref |  |
| Once or twice a week | 207 | (1·4) | 366 | (3·4) | 2·50 | (1·90-3·28) |
| Every day | 54 | (0·4) | 161 | (1·5) | 4·23 | (2·63-6·80) |
| Violence outside the family | 653 | (4·5) | 1181 | (11·0) | 1·07 | (0·81-1·41) |

*Source.* FRA Violence Against Women Survey dataset, 2012.

IPV=intimate partner violence. OR=odd-ratio. CI=confidence interval. MD=missing data.

When not specified, “No” is used as reference.

OR computed with binomial logistic regression models.

For each variable, the reference category is indicated as “Ref” (e.g. for age, the 18-29 age class is the category of reference for OR).

MD were considered as category.

Appendix J. STROBE Statement- Checklist of items that should be included in reports of cross-sectional studies

|  | Item No | Recommendation | Page No |
| --- | --- | --- | --- |
| **Title and abstract** | 1 | (*a*) Indicate the study’s design with a commonly used term in the title or the abstract | 1 |
|  |  | (*b*) Provide in the abstract an informative and balanced summary of what was done and what was found | 3 |
| Introduction | | | |
| Background/rationale | 2 | Explain the scientific background and rationale for the investigation being reported | 5 |
| Objectives | 3 | State specific objectives, including any prespecified hypotheses | 5 |
| Methods | | | |
| Study design | 4 | Present key elements of study design early in the paper | 5 |
| Setting | 5 | Describe the setting, locations, and relevant dates, including periods of recruitment, exposure, follow-up, and data collection | 5 |
| Participants | 6 | (*a*) Give the eligibility criteria, and the sources and methods of selection of participants | 5 |
| Variables | 7 | Clearly define all outcomes, exposures, predictors, potential confounders, and effect modifiers. Give diagnostic criteria, if applicable | 6 |
| Data sources/ measurement | 8* | For each variable of interest, give sources of data and details of methods of assessment (measurement). Describe comparability of assessment methods if there is more than one group | 6 |
| Bias | 9 | Describe any efforts to address potential sources of bias | 6 |
| Study size | 10 | Explain how the study size was arrived at |  |
| Quantitative variables | 11 | Explain how quantitative variables were handled in the analyses. If applicable, describe which groupings were chosen and why | 6 |
| Statistical methods | 12 | (*a*) Describe all statistical methods, including those used to control for confounding | 6 |
|  |  | (*b*) Describe any methods used to examine subgroups and interactions | 6 |
|  |  | (*c*) Explain how missing data were addressed | 6 |
|  |  | (*d*) If applicable, describe analytical methods taking account of sampling strategy |  |
|  |  | (*e*) Describe any sensitivity analyses |  |
| Results | | | |
| Participants | 13* | (a) Report numbers of individuals at each stage of study—eg numbers potentially eligible, examined for eligibility, confirmed eligible, included in the study, completing follow-up, and analysed | 6 |
|  |  | (b) Give reasons for non-participation at each stage | 6 |
|  |  | (c) Consider use of a flow diagram |  |
| Descriptive data | 14* | (a) Give characteristics of study participants (eg demographic, clinical, social) and information on exposures and potential confounders | 6 |
|  |  | (b) Indicate number of participants with missing data for each variable of interest | 6 |
| Outcome data | 15* | Report numbers of outcome events or summary measures | 9 |
| Main results | 16 | (*a*) Give unadjusted estimates and, if applicable, confounder-adjusted estimates and their precision (eg, 95% confidence interval). Make clear which confounders were adjusted for and why they were included | 9 |
|  |  | (*b*) Report category boundaries when continuous variables were categorized |  |
|  |  | (*c*) If relevant, consider translating estimates of relative risk into absolute risk for a meaningful time period |  |
| Other analyses | 17 | Report other analyses done—eg analyses of subgroups and interactions, and sensitivity analyses | 12 |
| Discussion | | | |
| Key results | 18 | Summarise key results with reference to study objectives | 13 |
| Limitations | 19 | Discuss limitations of the study, taking into account sources of potential bias or imprecision. Discuss both direction and magnitude of any potential bias | 13 |
| Interpretation | 20 | Give a cautious overall interpretation of results considering objectives, limitations, multiplicity of analyses, results from similar studies, and other relevant evidence | 13 |
| Generalisability | 21 | Discuss the generalisability (external validity) of the study results | 14 |
| Other information | | | |
| Funding | 22 | Give the source of funding and the role of the funders for the present study and, if applicable, for the original study on which the present article is based | 14 |

*Give information separately for exposed and unexposed groups.

Appendix K. Summary box

**Section 1: What is already known on this topic**

We did not do a formal systematic review because no uniform, general population-based study was conducted to date in intimate violence partner (IPV) among European women. We searched PubMed for previously published studies on IPV prevalence to date. We selected studies reporting population-based prevalence in a nonspecific population (46 articles). Many studies were related to specific populations such as pregnant women, HIV positive, older, or married women. Definition of IPV varied across studies and its measure was often partial i.e. considering only one or two dimensions among the physical, sexual, or psychological dimensions of IPV. Sexual and psychological IPV were often excluded from analyses. A limited number of potential determinants were considered, maximum eighteen at a time. The citations of included articles were also searched. The review was completed by grey literature analysis. We selected the WHO report on global and regional estimates of violence against women, which compiled and aggregated data from several studies.

**Section 2: What this study adds**

Our study proposes an estimation of IPV prevalence among ever-partnered women, grounded on data uniformly collected in a European population-based survey, and on a comprehensive definition of violence, similar to IPV definition from the International violence against women survey and the World Health Organization multi-country study on women’s health and domestic violence. We studied 27 factors as potential determinants for IPV covering demographic, socioeconomic, and health characteristics of IPV victims or survivors and of current partners of IPV victims in a large sample. We found an estimated prevalence of lifetime overall IPV i.e. physical, sexual, or psychological IPV, of 51·7% (CI95% 51·2-52·2) among European women having already been in a relationship. Psychological IPV was the most prevalent isolated reported type of IPV (29·6%). A total of 6·3% of women reported having experienced sexual, physical, and psychological IPV in their lifetime. Physical, respectively sexual IPV was about 3-fold, resp. about 4-fold less reported when the declared perpetrator was the current partner (resp. 7·0% for current partner vs 24·1% for previous partner and 2·3% vs 10·7%). Overall IPV lifetime prevalence is high among exposed European women. Several strongly associated factors with lifetime reported IPV as well as broad differences in prevalence according to the status of the perpetrator (current vs previous partner) suggest an even higher actual prevalence because of a probable under-reporting of IPV in currently partnered women. IPV was consistently associated with impaired self-perceived health or functioning and a higher likelihood of history of childhood adversities. Evidence from available population-based epidemiological studies on IPV emphasizes that individual data are of limited interest in terms of health policy and prevention. First, IPV is a matter of at least two individuals and should be considered as such, at the time of the conception of surveys and data collection. Characteristics of the current partner are consistently and strongly associated with all IPV dimensions. Characteristics of the relationship should also be considered. Interventions cannot be only women-centered. Second, time appears a crucial dimension in understanding and preventing IPV and their consequences. Meanwhile, its role is still unclear and makes data collection and interpretation uncertain. Lifetime reported IPV decreases with time which is counter-intuitive since exposition is cumulative and exposition cannot decrease with time, so cannot violence. Results suggest that IPV is a common phenomenon which is not uniformly distributed across populations. Nature, intensity, and concentration of IPV may be differentiated according to specific profiles of victims, partners, and couples. Trajectories or population segmentations in terms of IPV sequences and differentiated risk profiles are still to be identified and characterized. Two perspectives should be considered: undifferentiated and general educational interventions about IPV so that knowledge and culture may change over time in general population; differentiated and targeted interventions appropriate to risk level of IPV in women, current, and ex-partner. Special attention should be paid on the time for these interventions: interventions should be adapted to specific trajectories and following possible accelerations in violence sequences resulting in escalating intensity and frequency with death as ultimate endpoint. Finally, data collection should evolve and different approaches confronted so that we may diminish the risk of underreporting, e.g. confronting surveys based on face-to-face interviews, classical self-completed questionnaires, social networks signals and forum contents analysis, e-cohorts, and sub-population studies (e.g. forensic examinations of IPV victims or perpetrators in forensic units). Risk is high that many IPV and specific IPV victim’s profiles could remain invisible.
